# PfSPZ Vaccine induces focused humoral immune response in HIV positive and negative Tanzanian adults

**DOI:** 10.1016/j.ebiom.2024.105364

**Published:** 2024-09-30

**Authors:** Anneth Tumbo, Freia-Raphaella Lorenz, Annie S.P. Yang, Stephanie Sefried, Tobias Schindler, Maximilian Mpina, Jean-Pierre Dangy, Florence A. Milando, Mohammed A. Rashid, Gloria Nyaulingo, Kamaka Ramadhani, Said Jongo, Philip L. Felgner, Yonas Abebe, B. Kim Lee Sim, L.W. Preston Church, Thomas L. Richie, Peter F. Billingsley, Tooba Murshedkar, Stephen L. Hoffman, Salim Abdulla, Peter G. Kremsner, Benjamin Mordmüller, Claudia Daubenberger, Rolf Fendel

**Affiliations:** a Department of Medical Parasitology and Infection Biology, Swiss Tropical and Public Health Institute, Basel, Switzerland; b University of Basel, Basel, Switzerland; c Institute of Tropical Medicine, University of Tübingen, Tübingen, Germany; d Ifakara Health Institute, Bagamoyo Branch, Bagamoyo, United Republic of Tanzania; e University of California, Irvine, California, United States; f Sanaria Inc., Rockville, Maryland, United States; g Radboud Center for Infectious Diseases, Department of Medical Microbiology, Radboud University Medical Center, Nijmegen, the Netherlands; h German Center for Infection Research (DZIF), Partner Site Tübingen, Tübingen, Germany; i Centre de Recherches Médicales de Lambaréné, Lambaréné, Gabon

**Keywords:** HIV, PfSPZ Vaccine, *Plasmodium falciparum*, *Plasmodium falciparum* circumsporozoite protein, *Plasmodium falciparum* merozoite surface protein 5, Protein microarray

## Abstract

**Background:**

PfSPZ Vaccine, a promising pre-erythrocytic stage malaria vaccine candidate based on whole, radiation-attenuated *Plasmodium falciparum* (Pf) sporozoites (SPZ), has proven safe and effective in mediating sterile protection from malaria in malaria-naïve and exposed healthy adults. Vaccine-induced protection presumably depends on cellular responses to early parasite liver stages, but humoral immunity contributes.

**Methods:**

On custom-made Pf protein microarrays, we profiled IgG and IgM responses to PfSPZ Vaccine and subsequent homologous controlled human malaria infection (CHMI) in 21 Tanzanian adults with (n = 12) or without (n = 9) HIV infection. Expression of the main identified immunogens in the pre-erythrocytic parasite stage was verified by immunofluorescence detection using freshly purified PfSPZ and an *in vitro* model of primary human hepatocytes.

**Findings:**

Independent of HIV infection status, immunisation induced focused IgG and IgM responses to circumsporozoite surface protein (PfCSP) and merozoite surface protein 5 (PfMSP5). We show that PfMSP5 is detectable on the surface and in the apical complex of PfSPZ.

**Interpretation:**

Our data demonstrate that HIV infection does not affect the quantity of the total IgG and IgM antibody responses to PfCSP and PfMSP5 after immunization with PfSPZ Vaccine. PfMSP5 represents a highly immunogenic, so far underexplored, target for vaccine-induced antibodies in malaria pre-exposed volunteers.

**Funding:**

This work was supported by the Equatorial Guinea Malaria Vaccine Initiative (EGMVI), the Clinical Trial Platform of the German Center for Infection Research (TTU 03.702), the Swiss Government Excellence Scholarships for Foreign Scholars and Artists (grant 2016.0056) and the Interdisciplinary Center for Clinical Research doctoral program of the Tübingen University Hospital. The funders had no role in design, analysis, or reporting of this study.

**Research in context**

**Evidence before this study**

PfSPZ Vaccine, based on live, radiation-attenuated sporozoites, is a promising malaria vaccine candidate shown to induce protective immunity in healthy African adults. Protection is suggested to be mainly attributed to cellular immune responses, while the contribution of antibodies is not clear. Wide implementation of PfSPZ Vaccine in sub-Saharan Africa requires better understanding of immunogenicity in HIV-infected persons.

**Added value of this study**

We profiled individual antibody responses to PfSPZ Vaccine by protein microarray and detected no significant difference in the response patterns of HIV positive and negative Tanzanian adults. Independently of the HIV infection status, merozoite surface protein 5 (PfMSP5) was one of the main targets of vaccine-induced antibodies. We demonstrated that this antigen is expressed in the apical complex and on the surface of sporozoites.

**Implications of all the available evidence**

It has been shown that within the study, HIV negative individuals showed enhanced Inhibition of Sporozoite Invasion (ISI); nevertheless, our data suggests that HIV infection does not affect overall antibody levels to PfSPZ Vaccine. Additionally, PfMSP5 is an interesting candidate antigen in further malaria vaccine development, as this antigen is strongly recognized by PfSPZ Vaccine induced IgM antibodies.

## Introduction

Following decades of intensive efforts to reduce the global burden of malaria, the numbers of annual cases and malaria-associated deaths are similar to those in 2012.^1^ In 2022, an estimated 249 million malaria cases occurred worldwide causing 608,000 deaths.^2^ *Plasmodium falciparum* (Pf) is the deadliest malaria parasite, and by far the most prevalent in sub-Saharan Africa. Current control efforts rest on a combination of intensive vector control, rapid detection and treatment of infections.^3^ An effective malaria vaccine targeting Pf would be an important, complementary tool to facilitate malaria control and eventual elimination.^4^

Several vaccine development approaches are currently pursued that target the pre-erythrocytic stage of malaria, a bottleneck in the parasite life cycle composed of the sporozoite (SPZ) and liver stages. RTS,S/AS01, a subunit vaccine targeting the Pf circumsporozoite protein (PfCSP) is the first vaccine to be endorsed by WHO for use in children living in moderate to high malaria endemic regions.^5^ RTS,S/AS01 has shown a significant reduction in severe malaria cases yet fails to meet the goal of 75% vaccine efficacy against clinical malaria.^6^ Results of a phase 2b clinical trial in Burkina Faso as well as a phase 3 clinical trial in four countries (Burkina Faso, Mali, Kenya and Tanzania) investigating the vaccine candidate R21/Matrix-M, a biosimilar of the RTS,S vaccine with a different PfCSP stoichiometry and an alternative adjuvant, were published recently.^7^^,^^8^ In the phase 3 clinical trial, R21/Matrix-M mediated vaccine efficacy against clinical malaria was described as 75% at the seasonal sites and 67% at the perennial transmission sites over a follow-up period of 12 months. Both vaccines are now recommended to be used in national vaccination programs in several countries in Africa for use in young children.^9^ Nevertheless, there is an urgent need for further vaccines which can be applied also in adults and with higher vaccine efficacy, thus is able to prevent not only disease but also infection with Pf parasites per se. This is of high importance, as adults also significantly contribute to transmission and morbidity in high-transmission areas.^10^^,^^11^

An alternative vaccine approach rests on direct venous inoculation (DVI) of aseptic, purified, cryopreserved, metabolically active PfSPZ attenuated by either irradiation (PfSPZ Vaccine), genetic engineering (PfSPZ-GA1 and PfSPZ-LARC2 Vaccine) or concomitant anti-malaria drug treatment (PfSPZ-CVac).^12^^,^^13^ Vaccination with PfSPZ-CVac has provided up to 100% sterile protection for 10–12 weeks in malaria-naïve volunteers against both homologous (using the same Pf vaccine strain) and heterologous (using a genetically different Pf strain) Pf challenge administered as standardised controlled human malaria infection (CHMI).^14^, ^15^, ^16^ Live attenuated, whole organism based malaria vaccines expose the human immune system to a broad range of antigens with the potential to induce both cellular and humoral immunity mediating high levels of sterile protection against re-infection.^17^ For PfSPZ Vaccine, it is commonly accepted that the PfSPZ rapidly reach and infect hepatocytes but stop development at early liver stages due to radiation-induced DNA damage.^18^

A series of clinical trials has been published over the past years describing PfSPZ Vaccine immunogenicity, safety and protective efficacy against CHMI or field malaria exposure in sub-Saharan Africa.^19^, ^20^, ^21^ However, these trials were restricted to HIV negative individuals. Aiming at mass vaccination programs for malaria elimination, it is crucial to understand the interaction between HIV infection status and vaccination safety and outcome, as HIV prevalence varies between below 1% and up to 25% in some countries of sub-Saharan Africa.^22^^,^^23^ RTS,S/AS01 has been proven safe in HIV positive vaccinees^24^ but PfSPZ Vaccine as a live, yet non-replicating vaccine needs to be evaluated carefully in immunocompromised individuals.^25^

A randomised, double-blind, placebo-controlled clinical trial was recently conducted to evaluate the safety, immunogenicity, and protective efficacy against homologous CHMI of PfSPZ Vaccine administered as five doses, each 9.0 × 10^5^ PfSPZ, at study days 1, 3, 5, 7 and 29 to HIV negative and HIV positive volunteers compared to normal saline (NS) controls.^26^ It showed that 1) PfSPZ Vaccine was well tolerated and safe in HIV positive individuals, 2) sera from HIV negative vaccinees had significantly higher inhibition of PfSPZ invasion of hepatocytes *in vitro* and antibody-dependent complement deposition (ADCD) and Fcγ3B binding by anti-PfCSP and ADCD by anti–cell-traversal protein for ookinetes and SPZ (anti-PfCelTOS) antibodies, and 3) 80% of HIV negative and none of HIV positive vaccinees were protected against controlled human malaria infections.

Protein microarrays are an elegant tool to screen for antibodies against larger parts of a pathogen's proteome in a largely unbiased approach.^27^ Pf-specific arrays have been widely used to profile naturally acquired immunity against malaria,^28^, ^29^, ^30^ identifying markers of exposure^31^, ^32^, ^33^ or correlates of protection after natural exposure and vaccination.^34^, ^35^, ^36^ Serum samples were collected from study volunteers pre-and post-vaccination and post-CHMI to probe a custom-made protein microarray covering 228 Pf proteins. Our study aimed to compare IgG and IgM antibody profiles before and after immunisation with PfSPZ Vaccine in HIV positive and negative Tanzanian adults, as well as to study the impact of CHMI on antibody profiles. Specific antibody responses were confirmed using ELISA. To verify the expression of the PfSPZ Vaccine-induced immune-dominant antigens, we stained for these targets using an immunofluorescence assay (IFA) using freshly isolated sporozoites and Pf infected hepatocytes.

## Methods

### Study design and samples

**Fig. 1:**
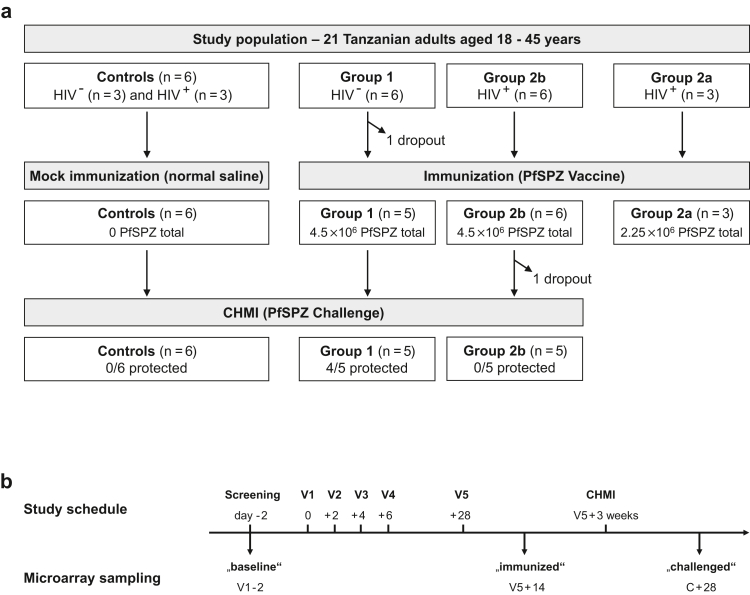
**Study design and sampling time points for microarray analysis**. (a) Volunteer group allocation. The trial included an HIV positive pilot group (group 2a) that received 4.5 × 10^5^ PfSPZ of PfSPZ Vaccine in each inoculation and did not undergo CHMI. Group 1 and group 2b comprised each six HIV negative and HIV positive volunteers that received five times 9 × 10^5^ of PfSPZ Vaccine in direct venous inoculation. The placebo control group had six volunteers that were HIV negative (n = 3) or HIV positive (n = 3). (b) Serum sample collection and study flow chart. Serum samples for microarray analysis were collected at baseline (V1–2), 14 days after the fifth injection (V5 + 14) and 28 days after CHMI (C + 28).

Study samples originate from a phase 1 clinical trial designed to test safety, immunogenicity and efficacy of PfSPZ Vaccine in 21 Tanzanian adults, including 12 HIV seropositive participants and 9 HIV seronegative participants.^26^ There were no predetermined objectives regarding male vs. female enrollment, and all female volunteers were required to complete a supplementary written informed consent form to verify that the volunteer understood that in order to participate in this study, one should neither be pregnant nor breastfeeding and take specific measures not to get pregnant for the duration of the trial. Sex of participants was self-reported and can be found in supplement 1 of the publication describing the clinical trial.^26^ HIV serology was performed using SD BIOLINE HIV-1/2 3.0 test (03FK10, Abbott diagnostics, Korea) and confirmed using the UniGold HIV test (1206502, Trinity Biotech, Ireland). All HIV positive subjects were on continuous anti-retroviral treatment and had a CD4+ T cell count above 500 cells/μl at screening visit. The study started with the HIV negative group (group 1), with blinded randomization to five immunisations of Sanaria® PfSPZ Vaccine with a dose of 9.0 × 10^5^ PfSPZ per injection (n = 6) or normal saline placebo (n = 3), both administered by direct venous inoculation (DVI) on study days 1, 3, 5, 7 and 29. There followed an open label HIV positive pilot group (group 2a) receiving 4.5 × 10^5^ PfSPZ of PfSPZ Vaccine on the same schedule (n = 3), followed after a three week stagger (a week before the day 29 immunization of pilot group) by a second blinded, randomized cohort of 9 HIV positive volunteers (group 2b), also divided between PfSPZ Vaccine (9.0 × 10^5^ PfSPZ per injection) (n = 6) or normal saline placebo (n = 3) administered on the same schedule. Efficacy was assessed via homologous CHMI of the two main groups (excluding the pilot group) three weeks after fifth dose. One HIV negative and one HIV positive vaccinee were excluded from the study during immunisation and challenge phase, respectively, resulting in a total of 5 participants in both groups undergoing CHMI. Out of these, 4/5 HIV negative vaccinees were sterilely protected following CHMI, while 5/5 HIV positive and 6/6 placebo recipients developed asexual blood stage parasitaemia (Fig. 1a). One of the 5 HIV positive individuals was detected to have a parasitaemia with a local strain during vaccination period, was treated, and received CHMI 3 weeks later.^26^

### Ethics

The study was conducted from February to August 2018 at the Ifakara Health Institute in Bagamoyo, Tanzania, and is registered at ClinicalTrials.gov as NCT03420053. The trial was approved by the Institutional Review Board for the Ifakara Health Institute (IHI-IRB) (ref No. IHI/IRB/No.22-2017), Tanzanian Food and Drug Administration (TFDA) (TFDA Auth. No. 0017/CTR/0016/6), Tanzanian National Institute for Medical Research (NIMR) (ref. NIMR/HQ/R.8a/Vol.IX/2642), the Ethical Committee of Northern and Central Switzerland (EKNZ), and the US Food and Drug Administration under an IND. Written informed consent was obtained from participants prior to enrolment. All trial procedures were conducted in accordance with good clinical practices (GCP) and under the Declaration of Helsinki.

### Serum sample collection and processing

Whole blood samples were collected in 10 ml blood collection tubes with clot activators (Becton Dickinson) and allowed to stand for 1 h at room temperature (RT) to facilitate serum formation followed by centrifugation at 2000*g* for 10 min. Serum was then collected, aliquoted and stored at −80 °C until analysis. Time points of serum samples taken from volunteers and analysed in this study included baseline (before first vaccine inoculation), 14 days past last vaccination and 28 days post CHMI.

### Protein microarray

Microarray slides were obtained from the University of California Irvine, Irvine, California, US.^29^ 262 protein fragments, comprising 228 defined Pf antigens were expressed in an *E. coli-*based cell-free *in vitro* system and printed onto 16-pad ONCYTE AVID® (PolyAn GmbH, Berlin, Germany) nitrocellulose slides. Expressed fragments ranged in size from 100 bp to 1800 bp and covered both full proteins and protein domains (Table S1). The antigens are a selection of frequently observed antigenic targets in previous larger scale microarray studies, depicting both naturally acquired immunity^37^^,^^38^ and vaccination trials,^29^^,^^30^^,^^35^ and were already successfully applied using samples obtained from individuals with acute disease, individuals from endemic areas or in PfSPZ-CVac vaccine trials.^16^^,^^39^

A multiplex assay allowed the screening for both IgG and IgM antibodies simultaneously.^40^ Serum samples were diluted 1:50 in 0.05x Super G Blocking Buffer (Grace Bio-Labs, Inc., Oregon, US), supplemented with 10% *E. coli* lysate (GenScript, Piscataway, New Jersey, US) and incubated for 30 min on a shaker at RT. The positive control, a pool of sera sampled in a malaria hyperendemic area in Ghana, and the negative control, serum of a malaria-naïve European donor, were treated likewise. Microarray slides were rehydrated at RT by addition of 100 μl 0.05x Super G blocking buffer per pad. After removal of the rehydration buffer, 100 μl/pad of diluted sera samples were added onto the slides and incubated overnight at 4 °C on an orbital shaker at 180 rpm. Serum dilutions were removed the following day and slides were washed three times using 1x TBST buffer (Grace Bio-Labs, Inc.). During each washing step, 200 μl wash buffer/pad was applied and slides were incubated for 5 min on the shaker. Secondary anti-IgG (goat anti-human IgG QDot™800, Grace Bio-Labs, Cat# 110635) and anti-IgM antibodies (biotin-SP-conjugated goat anti-human IgM, Jackson ImmunoResearch Labs Cat# 109-065-043, RRID:AB_2337625) were diluted 1:200 and 100 μl were added onto the slides. Following an incubation period of 2 h at RT on the shaker, slides were washed three times before application of 100 μl/pad of a tertiary Qdot™585 Streptavidin Conjugate (Invitrogen, Cat# Q10111MP) at a 1:250 dilution. Slides were incubated for 1 h at RT on the shaker, subsequently followed by another three washing steps before rinsing with (ultrapure) water and drying by centrifugation in 50 ml Falcon tubes at 500 g for 10 min. Images of arrays were taken in the ArrayCAM® Imaging System (Grace Bio-Labs) with an acquisition time of 4 s and an exposure time of 200 ms and 5 ms for IgG and IgM images, respectively. Median raw signal intensities of the single array spots and surrounding background areas were obtained using the ArrayCAM® 400-S Microarray Imager Software. All pictures were manually checked for correct recognition of spot locations and potential noise signals.

### Pre-processing of microarray data

Microarray data analysis was performed in R statistical software package version 3.6.2.^41^ Data pre-processing comprised the removal of noise signals, followed by background correction, data transformation and normalisation steps. Every spot intensity signal was corrected for local background reactivity by applying the normal-exponential convolution model^42^ (available in the LIMMA package)^43^ in combination with a saddle-point approximation for initial parameter estimation.^44^ The model assumes exponentially distributed true spot signals overlaid by normally distributed background signals and is suggested as preferred correction method to reduce variability in the low-intensity signal range.^42^ Resulting antigen signal intensities were log_2_-transformed to approach a normal distribution. Normalisation between arrays and thus adjustment for sample-dependent background reactivity to *E. coli* lysate was ensured by subtraction of the median signal intensity of mock expression spots on that particular array.

### Statistics

Antigens were defined as differentially recognized between the test groups if they yielded a p < 0.05 (using the Welch-corrected Student's t-test to take potential unequal variances into account) and a fold change >2 in mean signal intensities for the estimation of specific antibodies on the protein microarrays. Differential responses were classified as relevant if they further reached p < 0.05 following Benjamini-Hochberg (BH) correction for multiple testing and a high effect size (Hedge's g > 0.8). The effect of intervention (“placebo”, “vaccine”) and sample collection time point (“baseline”, “immunised”, “challenged”) on the measured signal intensity of single antigens was assessed using a two-way mixed ANOVA model.

An antigen-specific threshold for seropositivity was set at a signal intensity of 3 log_2_-levels above the median intensity in the naïve controls. The antibody breadth of every sample described the number of seropositive antigens at one particular time point. Baseline differences in antibody breadth between the HIV positive and negative study population were estimated by Wilcoxon-Mann-Whitney tests. The change in antibody breadth over immunisation and CHMI was assessed in an aligned rank transform ANOVA using the function provided in the “ARTool” package.^45^ Seroconversion within one participant was defined as exceeding the seropositivity threshold between two time points with signal increase of at least 2 log_2_-levels; seroreversion was defined as drop below the threshold with a signal decrease of 2 log_2_-levels. Signal fluctuations around the threshold with intensity changes smaller than 2 log_2_-levels within one sample at different time points were designated as borderline reactivity. Volcano plots were generated in Graph Pad Prism 8, all other plots in R using the ggplot2,^46^ gplots,^47^ ggbeeswarm,^48^ lemon^49^ and PAA packages.^50^

### Immunological assays

IgG antibodies to PfCSP as well as IgG and IgM antibodies to PfMSP5 were assessed quantitatively by ELISA as described before.^16^^,^^51^ For the estimation of IgG antibodies, 96-well plates (Costar 96 well microtiter high binding plates) were coated overnight at 4 °C either with 0.5 μg/ml of the recombinant PfCSP protein (sequence from PfNF54, Genbank: XP_001351122, amino acid 199–377, expressed in *E. coli*) in 100 μl coating buffer (0.1 M Sodium Bicarbonate, pH 9.6) or 0.5 μg/ml of recombinant PfMSP5 protein in 100 μl coating buffer. The sequence of PfMSP5 (amino acid 23–255) was retrieved from PlasmoDB (PF3D7_0206900.1), and the N-glycosylation site, which showed full jury agreement according to NetNGlyc 1.0, was removed during codon optimization (T194A). After expression in HEK293F cells, the antigen was tested on SDS-PAGE (Fig. S1), and the protein expression was confirmed by mass spectrometry analysis. After coating the ELISA plates with the antigen, they were washed three times with 1X PBS, 0.1% Tween 20 (PBST) and blocked with PBST supplemented with 5% bovine serum albumin (BSA) and 0.5 mM EDTA. Plates were washed three times. Subsequently, serial dilutions of serum samples (in blocking buffer) were added in duplicate and incubated at RT for 1 h. After three washes, peroxidase labelled goat anti-human IgG (Jackson ImmunoResearch, Cat# 109-035-098, RRID:AB_2337586) was added at a dilution of 1:10,000 and incubated at RT for 1 h. Plates were washed three times, TMB peroxidase substrate was added for plate development, and the plates were incubated for 10 min at RT. Reactions were stopped with 1 M hydrochloric acid (HCl) stop solution. For the estimation of PfMSP5 specific IgM antibodies, the same procedure was applied, with the exception that the blocking buffer was 1X RotiBlock (Roth) and the secondary antibody was HRP-conjugated goat anti-human IgM (ImmunoReagents, Cat# GtxHu-006-E2HRPX, RRID: AB_3624299) at a dilution of 1:5000. The plates were immediately read with a CLARIOstar microplate reader (BMG) at 450 nm. Data analysis was performed with Clariostar Software Version 5.40 R2 and MARS Data Analysis Software Version 3.31. A negative control (serum from a malaria-naïve individual) was included in all assays. Serum of a pool of individuals with anti-PfCSP and PfMSP5 antibodies was used as positive control. Antibody concentrations were estimated relative to dilution series of highly pure human IgG (Thermo Fisher Scientific Cat# 31154, RRID:AB_243591) or highly pure IgM (Sigma–Aldrich Cat# I8260, RRID:AB_1163621), which were precoated on the same plates. To calculate the relative IgG and IgM concentration, a four-parameter logistic curve was fitted to the results of IgG and IgM positive control standards and the respective concentrations in the sera using R statistical software package version 4.0.4. The results are given as AU. One AU corresponds to the signal of 1 μg/ml of coated highly purified human IgG or IgM antibody.

### Immunofluorescence assay for PfSPZ and liver stages

PfNF54 SPZ were harvested from infected *Anopheles stephensi* mosquitoes on day 17–21 post blood meal by homogenizing the salivary gland and counted using a haemocytometer. The immunofluorescence assay was performed in a solution of 250,000 PfSPZ for each antibody combination following a similar method as described by Tibúrcio, Yang.^52^ For the non-permeabilized samples, PfSPZ were fixed in 4% paraformaldehyde and stained with the mouse anti-PfCSP monoclonal antibody 2A10 (BEI Resources; NIAID Cat# MRA-183A, RRID:AB_3626318) at 1:1000 and rabbit Polyclonal anti- PfMSP5 antiserum (BEI Resources; NIAID Cat# MRA-320, RRID:AB_3626320, generated against *P. falciparum* strain B8, AA 147–207 of the MSP5 ORF) at 1:150 dilution for 1 h at RT. For permeabilized samples, the PfSPZ were also fixed at 4% paraformaldehyde, permeabilized in 1% Triton X-100 for 10 min and stained with anti-PfGAPDH (The European Malaria Reagent Repository Cat# 7.2, RRID:AB_3626324 at 1:15,000 dilution) and the polyclonal anti-PfMSP5 (BEI Resources; NIAID Cat# MRA-320, RRID:AB_3626320). Anti-mouse Alexa 488 (Thermo Fisher Scientific Cat# A-11029, RRID:AB_2534088) and anti-rabbit Alexa 594 (Thermo Fisher Scientific Cat# A-11037, RRID:AB_2534095) were incubated with the samples for 1 h at RT (1:200 dilution). All antibodies were diluted with 3% bovine serum albumin in PBS. The staining was completed with DAPI (Thermo Fisher Scientific Cat# D1306, 300 nM final concentration) for 1 h. The solution was spun down for 5 min at 10,000 g, resuspended in 5 μl of PBS and air dried onto cover slides. VectaShield (Vector Laboratories, Cat# H-1000) was added to the samples and coverslips were mounted on top.

The liver stage assay closely followed the method reported by Miyazaki et al.^53^ Briefly, PfSPZ were harvested from infected *A. stephensi* mosquitoes between day 17 and 21 post blood meal in William's B media (see Miyazaki et al.^53^ for the composition) and supplemented with 10% heat-inactivated human serum (HIHS). A total of 62,000 PfSPZ (multiplicity of infection (MOI) of 1:1) were added to the hepatocytes 48 h post plating and spun down at 100 g for 10 min without brakes. The cultures were incubated at 37 °C in an atmosphere of 5% CO_2_ for a further 3 h, before media was refreshed with William's B medium supplemented with 10% HIHS. The cultures were incubated at 37 °C in an atmosphere of 5% CO_2_ for seven days, with daily media refreshments. On day 7, the cultures were fixed in 4% PFA, permeabilized with 1% Triton X-100 and blocked in 3% BSA. Mouse anti-MSP1 (Sanaria Cat# AD223, RRID:AB_3644263), mouse anti-EXP2 (The European Malaria Reagent Repository Cat# 7.7, RRID:AB_3626326) and rabbit anti-PfMSP5 were diluted to 1:100, 1:1000 and 1:150 respectively in 3% BSA and incubated with the samples for 1 h at RT. Anti-mouse Alexa 488 (Thermo Fisher Scientific Cat# A-11029, RRID:AB_2534088) and anti-rabbit Alexa 594 (Thermo Fisher Scientific Cat# A-11037, RRID:AB_2534095) were incubated with the samples for 1 h at RT (1:200 dilution). Nuclear content was stained using DAPI at a final concentration of 300 nM. All images were acquired using the Zeiss LSM880 with Airyscan on the 63× objective at 8× zoom for PfSPZ or 2x zoom for liver stages.

### Role of the funding source

The funders had no role in study design, in the collection, analysis and interpretation of data, in the writing of the report and the decision to submit the paper for publication.

## Results

### Study population and serum sampling

Serum samples for protein microarray analyses were collected at three time points during the study, namely “baseline” (two days before first immunisation), “immunised” (14 days past the fifth immunisation) and “challenged” (28 days past CHMI; Fig. 1b). Baseline study samples were collected from the HIV positive groups (n = 12, including groups 2a, 2b and placebo recipients) and HIV negative groups (n = 9, including group 1 and placebo recipients, Fig. 1a). Analysis of later time points (after immunisation and challenge) focused on the three main study groups of placebo controls (n = 6), the higher-dosed HIV positive vaccine group 2b (n = 6 after immunisation, n = 5 after challenge) and the HIV negative vaccine group 1 (n = 5) (Fig. 1a). All volunteers included in the study had no asexual blood stage parasitaemia at screening visit and before CHMI, as measured by malaria thick blood smears (TBS) and quantitative PCR (qPCR). Details of the clinical study itself are elaborated in the corresponding clinical trial publication.^26^

### Baseline immunity of the HIV positive and HIV negative study population

**Fig. 2:**
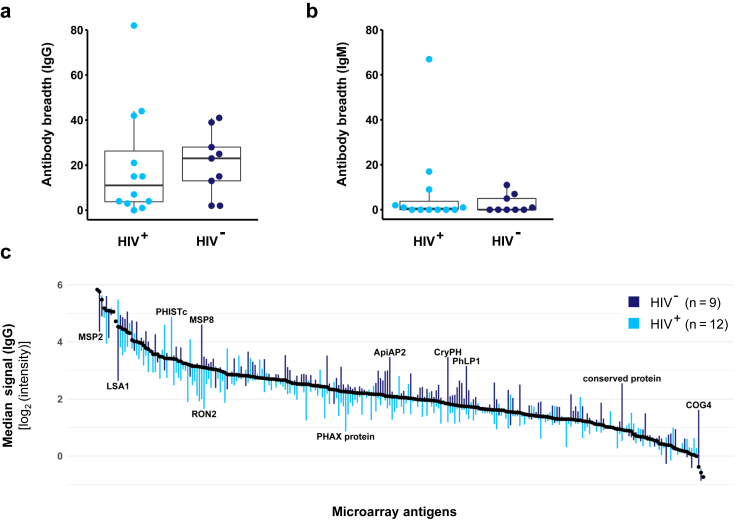
**Baseline immunity of study population stratified by HIV infection status**. Serum samples of HIV negative (n = 9) and HIV positive (n = 12) study participants were collected at baseline and probed on a protein microarray (see Table S1 for array design and antigen abbreviations). Individual breadths of IgG (a) and IgM (b) antibody responses were compared between HIV positive and HIV negative participants. Antibody breadth was defined as number of seropositive antigens exceeding a signal intensity threshold of 3 log_2_-levels above a malaria-naïve control. The boxplots give median antibody breadths, interquartile ranges (IQR) and whiskers of length 1.5 × IQR. (c) Antigens are sorted according to their overall median signal intensity, weighted for the different sizes of the HIV positive and HIV negative group. Bars give the deviation of the median signal intensities in the HIV negative (dark blue) and HIV positive group (light blue) from the overall median (see Table S2 list of antigens).

Malaria-specific humoral immunity was characterised by comparing the baseline microarray reactivity of HIV negative (n = 9) and HIV positive participants (n = 12). The number of individual Pf antigens recognized by IgG antibodies at baseline was highly variable between volunteers. Samples from HIV positive volunteers displayed a lower overall antibody breadth (median, range: 11, 0–82) compared to the HIV negative group (median, range: 23, 2–41) but no statistically significant differences were observed (p = 0.6, Wilcoxon-Mann-Whitney test, Fig. 2a). IgM reactivity was generally very low in both HIV positive (median, range: 0.5, 0–67) and negative subjects (median, range: 0, 0–11; Fig. 2b). When comparing the deviation of median signal intensities of samples from all HIV positive and negative volunteers from the overall weighted median intensity across all microarray antigens, both groups displayed a similar pattern of the IgG (Fig. 2c) and IgM responses (Fig. S2). Notably, IgG antibody levels against merozoite surface protein 2 (PfMSP2) and liver stage antigen 1 (PfLSA1) were lower in the HIV negative group, while antibody levels against merozoite surface protein 8 (PfMSP8) and several intracellular and core proteins (PfApiAp2, PfCryPH, PfPhLP1, PfCOG4, conserved protein PF3D7_0513200) were higher. HIV positive volunteers displayed an elevated recognition of an exported protein (PfPHISTc, PF3D7_0801000), but lower reactivity against a variety of proteins, including the rhoptry neck protein 2 (PfRON2) or a nucleoprotein (PHAX domain-containing protein, PF3D7_1021900). Highest overall IgG baseline responses were observed against some well-described markers of exposure^29^^,^^35^^,^^37^^,^^38^^,^^54^ such as liver stage antigen 3 (PfLSA3) or merozoite surface protein 2 (PfMSP2) but also against functionally uncharacterised proteins (Table S2).

### Antibody response induced by PfSPZ Vaccine immunisation and association with protection

**Fig. 3:**
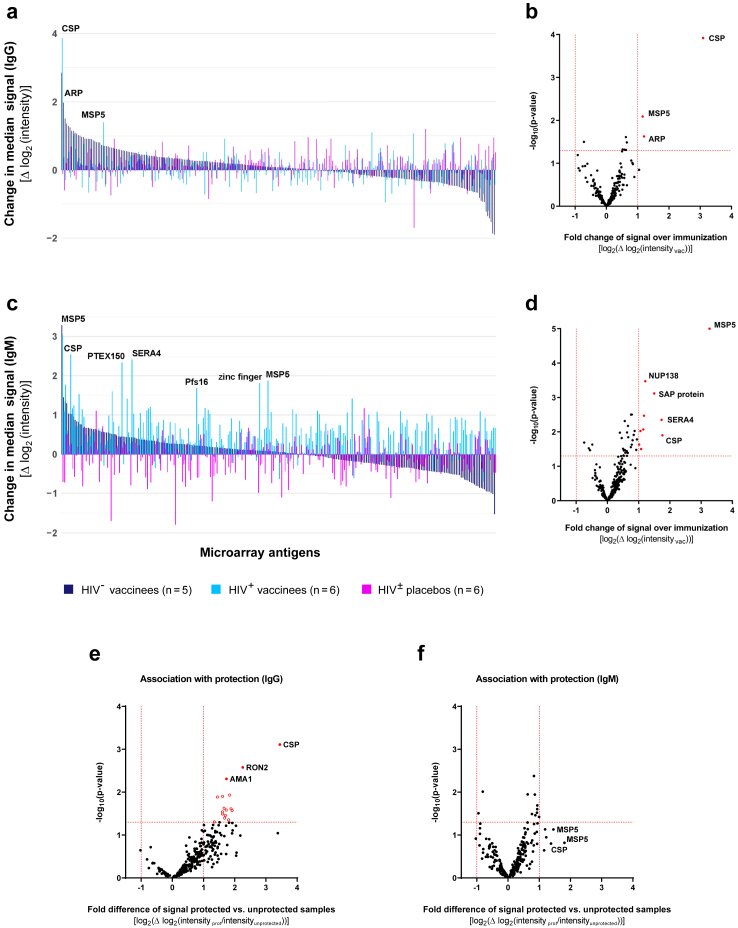
**Antibody responses aftervaccination usingPfSPZVaccine**. To identify vaccination-induced antibodies, microarray reactivity in samples collected after the completed immunisation phase were compared to their individual baseline reactivity. (a, c) Median changes in IgG (a) and IgM (c) signal intensities across 262 microarray antigens over the immunisation phase were assessed amongst placebo recipients (n = 6), HIV negative (n = 5) and HIV positive vaccinees (n = 6). Antigens are sorted by median intensity changes in the HIV negative vaccinees´ group. (b, d) Volcano plots of fold change in IgG (b) and IgM (d) and p-values (paired Student's t-test) of average signal intensity in all vaccinees (n = 11) compared to their baseline for all microarray antigens. In addition, antibodies correlating with sterile protection (protected group: n = 4; unprotected group: n = 12) against CHMI were analysed for both IgG (e) and IgM (f). Differentially recognized antigens (p-value <0.05 and fold change >2) are depicted in red (see Table S1 for antigen abbreviations and Table S3–S8 for summary of data).

The humoral immune responses after five DVIs of PfSPZ Vaccine were investigated next. Changes in specific reactivity to antigens between baseline and after immunisation were assessed amongst placebo recipients (n = 6), HIV negative vaccinees (n = 5, study group 1) and HIV positive vaccinees (n = 6, group 2b). As expected, in the placebo group, limited changes in median signal intensities were observed between these two time points, with few median signal changes exceeding 1 and none exceeding 2 log_2-_-levels (Fig. 3a). Irrespective of the vaccinees´ HIV status, immunisation induced significant IgG reactivity against PfCSP, PfMSP5 and a conserved asparagine-rich protein of unknown function (PfARP, PF3D7_0108300) with a mean fold change >2 and p < 0.05 (uncorrected paired Welch-corrected student's t-test) (Fig. 3a and b). A statistically significant increase after vaccination was observed for anti-PfCSP-IgG (Benjamini-Hochberg (BH) adjusted p = 0.03, Hedge's g = 1.40). Data stratified by HIV status of volunteers is given in Fig. S3a and b.

For IgM, the highest increase in signal intensity was observed for PfMSP5 in both groups of vaccinees. Additional changes in IgM antibody profile appeared amongst the HIV positive vaccinees, including responses to several pre-erythrocytic stage antigens like PfCSP, serine repeat antigen 4 (PfSERA4) and the parasitophorous vacuole membrane protein Pfs16, the liver stage protein translocon component PfPTEX150 and a zinc finger protein (PF3D7_1208800; Fig. 3c). Further reactive antigens observed amongst vaccinees were nucleoporin PfNUP138 and an SAP domain-containing protein (PF3D7_0912500; Fig. 3d). The strongest, statistically significant vaccination-induced IgM response was detected against PfMSP5 (BH adjusted p = 0.003, Hedge's g = 1.9). Data stratified by HIV status of the volunteers are provided in Fig. S3c and d.

PfSPZ Vaccine-induced anti-PfCSP-IgG and anti-PfMSP5-IgM responses were also observed in the three HIV positive vaccinees of study group 2a, receiving 4.5 × 10^5^ PfSPZ per injection (Fig. S4 for effects of different vaccine doses).

In addition, the humoral immune response was analysed for its association with protection against infection induced by challenge with CHMI. Across all study groups receiving the challenge (placebo recipients, group 1 and 2b), absolute antibody signal intensities after immunisation were compared between protected (n = 4) and unprotected participants (n = 12). Our results demonstrate that a specific set of antibodies is indeed correlated with protection against infection. Among protected individuals, the IgG antibodies with the highest reactivity were specific to PfCSP, PfRON2, and PfAMA1, while for IgM antibodies, the highest reactivity was detectable for PfCSP and PfMSP5 (Fig. 3e–f). The complete lists of antigens ranked according to their association with protection are provided in the supplements (Tables S7 and S8).

### Antibody response induced by homologous controlled human malaria infection

**Fig. 4:**
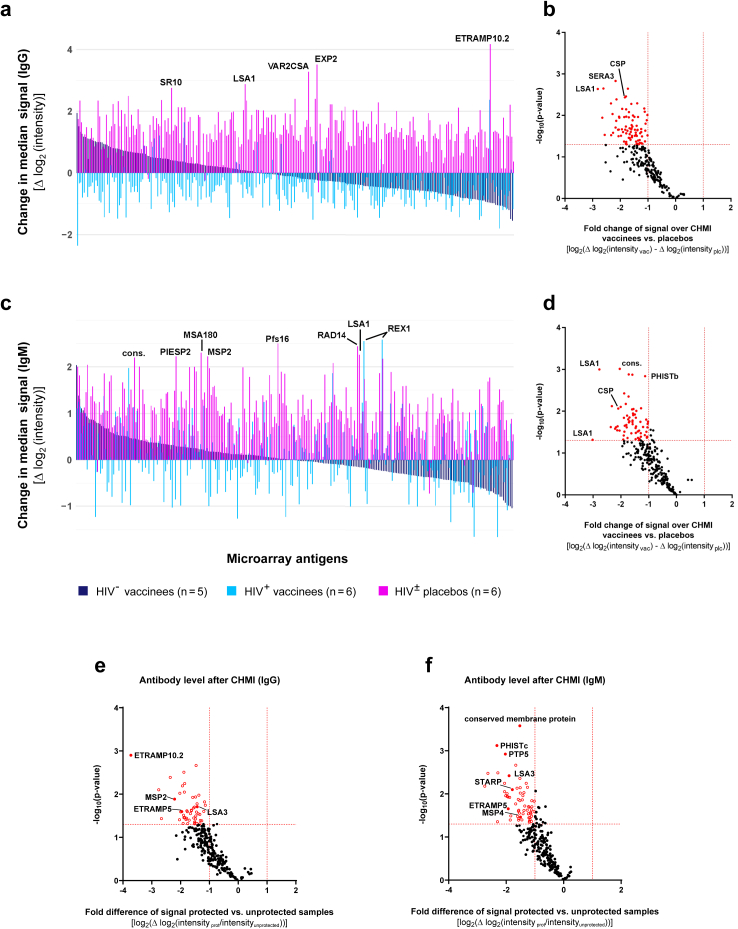
**Changes in antibody responses after homologous challenge**. Changes of microarray reactivity due to CHMI were identified by comparing signal intensities in samples collected before and four weeks after inoculation. Study participants were grouped into placebos (n = 6) and recipients of PfSPZ Vaccine (n = 10) with both groups containing equal numbers of HIV positive and HIV negative subjects. (a, c) Median changes of IgG (a) and IgM (c) signal intensities over challenge given for the 262 microarray antigens. Antigens are sorted by median intensity changes in the vaccinees´ group. (b, d) Volcano plot of mean changes in microarray IgG (b) and IgM (d) signal intensities over CHMI in the vaccinees compared to placebo group, as well as in the protected individuals (n = 4) compared to the unprotected individuals (n = 12) for IgG (e) and IgM (f). Fold change and p-values (Welch-corrected Student's t-test) are given for all microarray antigens. Significantly differentially recognized reactive antigens (p-value <0.05 and fold change >2) are depicted in red (see Table S1 for antigen abbreviations and Table S9–S12 for summary of data).

Sera of vaccinees (HIV negative (n = 5), HIV positive (n = 5)) and placebo recipients (n = 6) collected four weeks after CHMI display fundamentally different antibody profiles. Comparing the median change of IgG signal intensities before and after the CHMI across all microarray antigens, no significant booster effect of the PfSPZ Vaccine-induced IgG was seen in vaccinees but rather a decline in most antibody levels. In contrast, placebo recipients display a very broad antibody response to many antigens after CHMI. Strongest signal increases were observed in placebos for the early transcribed membrane protein PfETRAMP10.2, an exported protein (PfEXP2), the Pf erythrocyte membrane protein VAR2CSA and the liver stage proteins PfLSA1 and serpentine receptor PfSR10 (Fig. 4a). Comparisons of mean changes of IgG signal intensities before and after the challenge in all vaccinees vs. placebo recipients revealed a paucity of increased reactive antigens in vaccinees compared to many antigens recognized in placebos (mean absolute fold change >2 and p < 0.05 in Welch-corrected student's t-test). PfLSA1, the serine repeat antigen 3 (PfSERA3) and PfCSP were amongst the antigens differing most strongly between placebos and vaccines before and after CHMI (Fig. 4b). However, differences in signal intensities were not statistically significant following Benjamini-Hochberg (BH) adjustment but a strong effect with Hedge's g > 0.8 was seen for almost all identified reactive antigens (Fig. 4b).

Similarly, changes in the IgM antibody profile before and after CHMI in placebos with median signal increases >2 log_2_-levels were observed for a variety of blood stage antigens (including the merozoite surface proteins PfMSP2 and PfMSA180, the ring exported protein PfREX1 and the parasite-infected erythrocyte surface protein PfPIESP2) alongside with PfLSA1, the DNA repair antigen PfRAD14, the parasitophorous vacuole membrane protein S16 (Pfs16) and an uncharacterised conserved protein (PF3D7_0407700; Fig. 4c). In direct comparison of mean signal intensity changes between vaccinees and placebos, no malaria-specific antibody was elevated in the vaccinated group compared to placebo recipients. In stark contrast to this, the placebo group displayed a broad range of reactive antigens with particularly strong differences seen in various PfLSA1 fragments, PfCSP, exported proteins (including the PHISTb family member PF3D7_0532300) or a conserved protein (PF3D7_0817300; Fig. 4d). Performing the same analysis in protected vs. non-protected individuals, it becomes apparent that following the CHMI, antibody responses against a broad range of antigens were elicited based on the boosted immune response from transient parasitaemia. IgG as well as IgM antibodies specific to both pre-erythrocytic as well as erythrocytic stage strongly increased in the non-protected individuals (Fig. 4e–f).

### Kinetics of PfCSP and PfMSP5 specific antibody responses

**Fig. 5:**
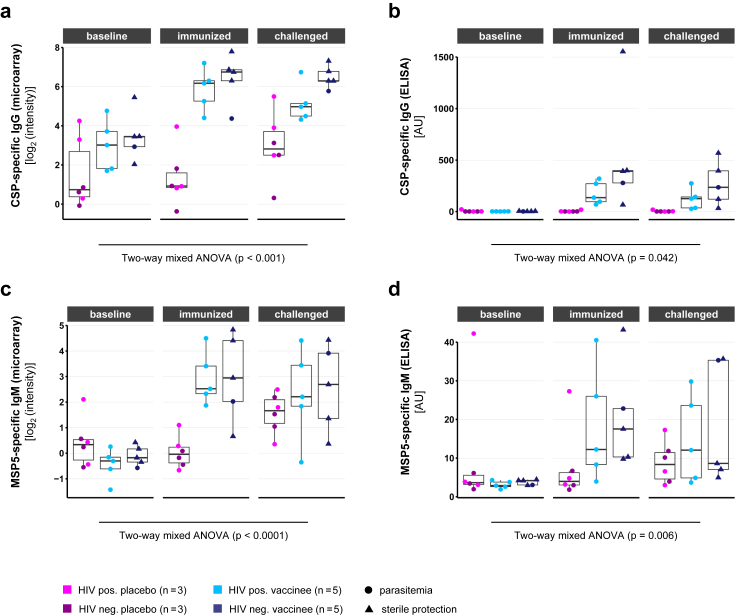
**Antibody kinetics of vaccine-induced antibodies**. Individual protein microarray and quantitative ELISA for anti-PfCSP-IgG (a, b) as well as for anti-PfMSP5-IgM (c, d) results are compared at baseline, 14 days after immunisation and 28 days after challenge. Protein microarray data are represented as normalised, log_2_-transformed signal intensities (a, c). Antibody concentrations were estimated quantitatively by ELISA in comparison to human IgG and IgM controls, respectively (b, d). Samples included placebos (n = 6), HIV positive (n = 5) and HIV negative vaccinees (n = 5). The influence of intervention group and sampling time point on the measured microarray antigen signal intensity was evaluated using a two-way mixed ANOVA model. The boxplots give median antibody breadths, interquartile ranges (IQR) and whiskers of length 1.5 × IQR.

Next, we assessed the changes in antibody levels against PfCSP and PfMSP5 before and after vaccination as well as after CHMI when measured by microarray or quantitative ELISA. Effects of the intervention group (“placebo” and “vaccine”) and sample collection time point (“baseline”, “immunised” and “challenged”) on the measured antibody levels were evaluated using a two-way mixed ANOVA model (Fig. 5).

For PfCSP-specific IgG measured by microarray, a low baseline reactivity was detected in all study groups (median microarray signal intensities <4 log_2_-levels). Following immunisation, both HIV positive and HIV negative vaccinees displayed elevated signals (median intensities >6 log_2_-levels) compared to the placebo group. CHMI induced no further increase in signal intensities amongst vaccinees but elevated antibody levels in placebo recipients, resulting in reduced group differences. ANOVA revealed a significant interaction between the effects of group and time point on the measured level of PfCSP-specific IgG (F(2, 28) = 10.14, p < 0.001) with significant simple main effects for both factors (p < 0.0001 each) (Fig. 5a).

The quantification of PfCSP-binding IgG by ELISA confirmed low baseline reactivity in placebo recipients and vaccinees (median below 2 AU) and an increase in antibody levels following the immunisation phase in vaccinees only (median level of 275 AU [95% confidence interval (CI): 69–399 AU]). Interestingly, PfCSP-specific IgG levels were higher in HIV negative study participants (median level of 392 AU [95% confidence interval (CI): 66–1554 AU]) in comparison to HIV positive study participants (median level of 136 AU [95% confidence interval (CI): 69–320 AU]), even though the difference was not statistically significant (Student's test of log-normalized data: p = 0.20). In contrast to the microarray analysis, the placebo recipients remained negative for PfCSP-specific IgG after CHMI, while the antibody levels in the vaccination group decreased slightly (median IgG concentration: 135 AU [95% CI: 32–396 AU]). Overall, the ANOVA showed significant interaction of time and group (F(2, 28) = 3.57, p = 0.042), with significant simple main effects for both group (p = 0.046) and the time factor (p = 0.042) (Fig. 5b).

IgM antibody levels against PfMSP5 followed a similar kinetic. In the microarray analysis, almost no reactivity at baseline was detected in any of the study groups (median signal intensities close to zero). Placebo recipients maintained a low reactivity throughout the immunisation phase, whereas both HIV positive and HIV negative vaccinees strongly responded to PfSPZ vaccination with increased PfMSP5-binding antibodies. Notably, antibody levels of placebo recipients approached those of vaccinees after CHMI (median signal intensities around 2 log_2_-levels). The interaction of intervention group and time point was highly significant in a two-way mixed ANOVA model (F(2, 28) = 13.38, p < 0.0001), with significant simple main effects for both intervention group (p = 0.026) and time (p < 0.0001) (Fig. 5c). The ELISA confirmed the microarray results of low baseline reactivity for placebo group and vaccinees below 4 AU. While the median concentration in the placebo group remained below 4 AU after the vaccination phase, median levels increased more than four-fold to 15 AU [95% CI: 8–41 AU] in vaccinees. After CHMI, the antibody level within the vaccination group decreased to 10 AU [95% CI: 5–35 AU]. Notably, the antibody concentration in the placebo group increased after challenge, reaching median levels of 8 AU [3–17 AU]. The two-way mixed ANOVA model revealed a significant interaction of group and time (F(2, 28) = 6.11, p = 0.006), with a significant simple main effect for time (p = 0.04), but not for the intervention group (p = 0.4) (Fig. 5d). Interestingly, PfMSP5-specific IgG was less prominently induced by the vaccine (Fig. S5). Whereas an increase in PfMSP5-specific IgG levels was detectable in all vaccinees by microarray (Fig. S5a), only part of the measured individuals developed increased IgG levels after vaccination in comparison to baseline as estimated by ELISA (Fig. S5b). The kinetics of other PfSPZ vaccination-induced antibodies, as identified in Fig. 3b and d, are given in Fig. S6. In summary, both microarray analysis and quantitative ELISA measurements confirmed that PfCSP and PfMSP5 are immunodominant antigens of PfSPZ vaccination irrespective of the HIV infection status of the volunteers.

### Immunofluorescence assay

**Fig. 6:**
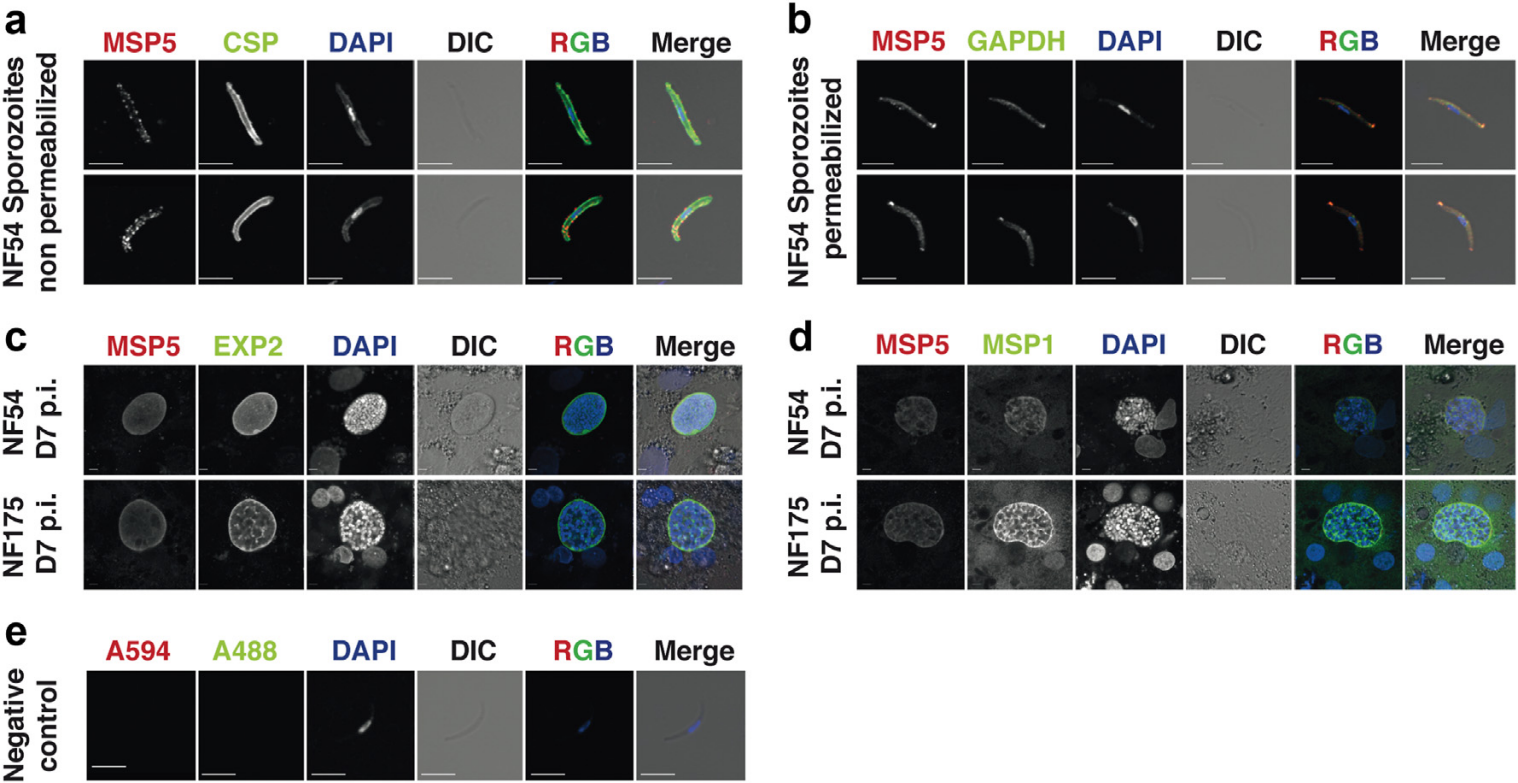
**Immunofluorescence of PfMSP5 on pre-erythrocytic stages**. Non-permeabilized (a) and permeabilized (b) PfNF54 PfSPZ were stained with rabbit polyclonal antibodies against PfMSP5, the nuclear stain DAPI, and either PfCSP or PfGAPDH respectively. Liver stage forms of PfNF54 and PfNF175 (7 days post invasion) were stained with PfMSP5 and PfEXP2 (c) and PfMSP5 and PfMSP1 (d). The negative control against the secondary antibody used for the PfMSP5 antibodies is shown in (e). Scale bar of 5 microns.

We have shown here that significant levels of PfMSP5-specific antibodies were induced after immunisation with PfSPZ Vaccine by microarray analysis and ELISA. Therefore, we conducted an immunofluorescence assay (IFA) to understand the subcellular localisation of PfMSP5 in PfSPZ and late liver stage parasites. Live PfSPZ extracted from *A. stephensi* salivary glands were co-stained with antibodies specific for PfCSP (clone 2A10) and PfMSP5 (polyclonal rabbit antiserum). The IFA showed the typical circumferential staining for PfCSP, as well as a punctiform staining on the PfSPZ surface for PfMSP5 (Fig. 6a). After permeabilization, the antibody staining for PfMSP5 was very prominent at the apical end of the parasite, suggesting expression in the rhoptries (Fig. 6b). IFA was also performed on *in vitro* cultivated late liver stage parasites (stained day 7 post invasion) using two different parasite strains: PfNF54 (PfSPZ Vaccine strain) and PfNF175 (West African strain with superior infectivity and maturation *in vitro*).^55^ In both strains, low expression levels of PfMSP5 were observed that did not seem to co-localize with staining for PfEXP2 (Fig. 6c) or PfMSP1, a well-known merozoite surface protein in later liver stage (Fig. 6d). Negative control IFA, performed as IFA without addition of primary antibodies, confirmed specificity of the secondary antibodies used (Fig. 6e).

### Acquisition of novel antibodies during immunisation and challenge

**Fig. 7:**
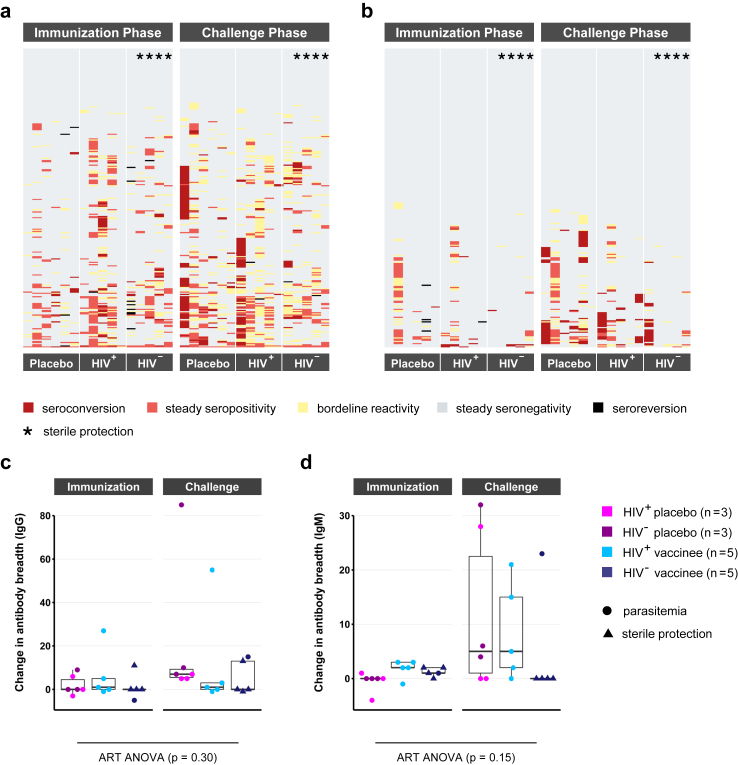
**Antibody acquisition and change in antibody breadth**. Changes in antibody repertoire over the immunisation and challenge phase were compared between placebo recipients (n = 6), HIV positive (n = 5) and HIV negative vaccinees (n = 5). The individual antibody breadth gives the number of seropositive antigens at a certain time point (signal intensity of >3 log_2_-levels above a malaria-naïve control). Seroconversion, the acquisition of a novel antigen, was defined as exceeding the seropositivity threshold accompanied by a signal increase of >2 log_2_-levels between the time points compared. Seroreversion was defined as drop below the threshold with a signal decrease of >2 log_2_-levels. Smaller signal fluctuations around the threshold were designated as borderline reactivity. (a, b) Changes in antigen recognition after immunisation and challenge are shown as heatmap for IgG (a) and IgM (b) with antigens depicted in rows and samples in columns. (c, d) Changes in the IgG (c) and IgM (d) antibody breadth over immunisation and challenge are compared between the three study groups of placebos, HIV positive and negative vaccinees. The influence of study group and phase on the change in antibody breadth was evaluated using an Aligned Rank Transform (ART) ANOVA model. The boxplots give median antibody breadths, interquartile ranges (IQR) and whiskers of length 1.5 × IQR.

Acquisition of novel antibody specificities following immunisation and CHMI are presented for each volunteer individually (Fig. 7a and b). An antigen-specific threshold of seropositivity was set at 3 log_2_-levels above the signal of a malaria-naïve control serum. Seroconversion, the acquisition of a novel antibody signal, was defined as a signal exceeding the seropositivity threshold together with an increase of intensity >2 log_2_-levels between the two time points compared. Seroreversion, the disappearance of an antibody signal, was defined as a decrease of signal intensity >2 log_2_-levels. Borderline reactivity was defined as signal fluctuations around the seropositivity threshold with intensity changes <2 log_2_-levels between the time points compared.

The majority of antigens spotted on the microarray were not recognized by the serum of the study participants at any of the time points assessed (Fig. 7a and b). Some antigens were recognized at baseline but remained unchanged upon immunisation using PfSPZ Vaccine and CHMI, while a selected number of antigens showed seroreversion. As already seen in Fig. 3, only few novel IgG or IgM antibody specificities were acquired during the immunisation. Comparing serum reactivity before and after CHMI, highly diverse responses were observed between the individuals tested (Fig. 7a and b). Some participants barely reacted to the challenge while others, all of whom developed asexual blood stage parasitaemia after CHMI (non-protected), acquired a broad range of novel antibody reactivities. In summary, a highly personalised IgG and IgM antibody profile was observed at baseline in these volunteers that remained mostly unchanged after PfSPZ Vaccine inoculation. Changes in antibody profiles were found in volunteers that developed asexual blood stage parasitaemia during CHMI.

Next, we measured the antibody breadth defined as the number of seropositive antigens in a sample and volunteer at a certain time point (Fig. 7c and d). As expected, few changes in the IgG or IgM breadth were observed in both placebos and vaccinees over the immunisation phase. CHMI in contrast induced a considerable expansion of antibody specificities in participants developing asexual blood stage parasitaemia (median of 5.5 and 6 novel antigens recognized for IgG and IgM, respectively), while protected subjects did barely respond at all (median of zero novel antigens recognized by both IgG and IgM). In accordance with these highly personalised humoral immune responses, no general impact of intervention phase (“immunisation” or “challenge”) and group (“placebo” or “vaccine”) on the IgG or IgM antibody repertoire could be proven in an aligned ranked transformed ANOVA model. The interaction effect for group and study phase on the change in antibody breadth was determined as p = 0.3 for IgG (F(1, 28) = 0.83) and p = 0.15 for IgM (F(1, 28) = 4.33).

## Discussion

We report a comparative proteomic analysis of serum samples collected from HIV positive and negative volunteers in a malaria-endemic area in Tanzania, undergoing either immunisation with radiation-attenuated PfSPZ (PfSPZ Vaccine) or placebo inoculation followed by homologous CHMI. IgG and IgM antibody profiles were assessed at baseline, two weeks after last immunisation and one month after CHMI on a protein microarray comprising 262 selected *P. falciparum* antigen fragments.

At baseline, serum samples of both the HIV positive and negative study population displayed highly personalised Pf-specific IgG repertoires or “fingerprints”^32^ with both groups recognizing a limited number of antigens. Coinfections of HIV and malaria were previously associated with reduced antibody response to an array of Pf antigens.^56^^,^^57^ In this study however, no obvious differences were detected in the Pf-specific antibody responses of HIV positive vs. HIV negative participants. A tendency of increased IgG responses against several liver and blood stage antigens including PfLSA1, PfMSP2, PfMSP4, PfMSP11, PfMSA180 and PfPHISTc were measured in HIV positives. IgG directed against PfMSP2 has been described as a marker of recent malaria exposure before,^31^ pointing towards a higher pre-exposure level due to increased malaria susceptibility in the HIV positive study population.^58^^,^^59^ As expected in a non-hyperendemic area, IgM baseline reactivity was hardly detectable in any of the groups.

Study participants assessed received either a normal saline inoculation or five doses of 9 × 10^5^ PfSPZ of PfSPZ Vaccine. Radiation-attenuated PfSPZ efficiently invade hepatocytes but stop development in early liver stages.^60^ In accordance to previously published results, our data confirm that immunization with PfSPZ Vaccine does not considerably change the pattern of humoral immune reactivity but rather maintains the personal immune “fingerprints”. Despite the broad range of potential antigenic targets presented on whole PfSPZ, immunization with the radiation-attenuated PfSPZ Vaccine, which is non-replicating, only induces a limited range of Pf-specific antibodies, as compared to immunization with the chemo-attenuated PfSPZ-CVac (CQ), which is fully replicating.^14^^,^^29^^,^^32^^,^^61^ These particularly include anti-PfCSP-IgG and anti-PfMSP5-IgM, both displaying comparably low baseline reactivity and a sharp increase in signal intensities amongst all vaccinees, regardless of their HIV infection status.

PfCSP, the target protein of the RTS,S/AS01 subunit vaccine, is also the immunodominant antigen after PfSPZ Vaccine inoculation, inducing high levels of specific IgG.^32^^,^^62^ Despite the evidence that some of these anti-PfCSP antibodies are indeed functional if directed against certain PfCSP epitopes, their overall contribution to protection induced by PfSPZ Vaccine remains unknown.^21^^,^^26^^,^^61^^,^^63^ Interestingly, a recent study in PfSPZ Vaccine immunised Tanzanians focusing on PfCSP-specific IgM found that these antibodies efficiently activate complement and block PfSPZ invasion of hepatocytes *in vitro*, supporting the potential functional relevance of PfCSP-specific IgM in vaccine induced protection.^64^ Similarly sera from protected individuals had higher inhibition of PfSPZ invasion.^26^

All vaccinees developed IgM against PfMSP5 following immunisation when measured by microarray and ELISA, and microarray data revealed an increase of PfMSP5-specific IgG for all individuals, whereas the ELISA suggest an increase only in a subpopulation (2 HIV negative individuals, and only in 1 HIV positive individual who was also infected by a local strain during the vaccination period). The *msp5* gene codes for a 272-residue protein with a C-terminal EGF-like domain and a GPI attachment motif.^65^ PfMSP5 is located on the surface of merozoites and seems to be highly conserved.^66^ So far, IgG against PfMSP5 was commonly known to correlate with naturally acquired immunity and was associated with protection from parasitaemia in a malaria mouse model^67^^,^^68^ and from clinical malaria in the field,^69^^,^^70^ qualifying PfMSP5 as potential candidate for blood stage malaria vaccines.^71^ However, the biological function of PfMSP5 appears to be dispensable for intra-erythrocytic parasite survival since viable knockout mutants have been described.^72^

Notably, IgG antibodies binding PfMSP5 have been detected and described in recipients of PfSPZ Vaccine and PfSPZ-CVac before.^14^^,^^73^ Recently, it was proposed that cross-reacting PfCSP-specific antibodies might account for increased PfMSP5 reactivity observed in a large scale protein microarray analysis following RTS,S/AS01E vaccination.^74^^,^^75^ Such an underlying phenomenon cannot be excluded here; however, quantitative ELISA using recombinantly expressed and purified PfMSP5 strongly supported the microarray results, indicating a specific binding of vaccine-induced IgG and especially IgM to PfMSP5. Furthermore, both published proteomic and transcriptomic studies suggested the potential expression of PfMSP5 not only during the blood stage, but already in the salivary gland PfSPZ stage,^76^, ^77^, ^78^ rendering it a potential antigenic target of any PfSPZ vaccine.

Here, using immunofluorescence detection, we demonstrated that PfMSP5 indeed seems to be expressed on the surface of PfSPZ. In permeabilized PfSPZ, PfMSP5 localizes within the apical end, suggesting its potential association with rhoptries. Across the PfSPZ surface, PfMSP5 occurs in a punctiform distribution, possibly integrated in lipid rafts, and potentially positioned above the PfCSP layer. A similar pattern was observed for PLP1 and SPECT, two PfSPZ surface proteins required for hepatocyte cell traversal.^79^ This may suggest an involvement of PfMSP5 in a similar process rather than in liver cell invasion or maturation. Cell traversal is essential for parasites to escape skin and blood vessels before reaching the liver. Traversal-deficient PfSPZ may still be capable of invading hepatocytes and maturation *in vitro* but fail to establish an infection under natural conditions.^79^ In contrast, during late liver stage development, PfMSP5 seemed to be expressed at low levels in two different parasite strains. Its lack of co-localisation with PfMSP1, a critical component of erythrocyte-binding complexes,^80^ in addition to its dispensability during blood stage development based on knockout parasites^72^ might point to a biological function during PfSPZ and/or liver stage. Previous studies primarily focused on the monitoring of PfSPZ Vaccine-induced IgG responses. However, there is growing awareness that Pf-specific antibodies of other subclasses, especially IgM, might contribute to malaria immunity and require further study. Our results indeed suggest that even though both IgG and IgM directed against PfMSP5 are found, the IgM response is more pronounced. Recently, Boyle et al. found merozoite-specific IgM, including antibodies targeting PfMSP5, to be rapidly acquired following CHMI in both malaria-naïve and pre-exposed persons. Longevity and kinetics of decay did not substantially differ from the respective IgG response, but merozoite-specific IgM proved considerably more effective in complement activation and high serum titers were associated with protection from clinical malaria.^81^ This also deserves particular consideration in the context of PfSPZ Vaccine, since efficient complement activation is essential in protective immunity against PfSPZ.^82^ Furthermore, induction of optimal protective efficacy has been determined to require 3 doses of 9 × 10^5^ PfSPZ^83^ suggesting that a significant number of parasites might not reach the liver but the spleen. Splenic marginal B-zones contain a special subtype of B-cells allowing a rapid, T-cell independent IgM response to systemic infections including malaria.^84^ Both T-cell-dependent and independent IgM-secreting B-cells have lately been reported to undergo somatic hypermutation following Pf infection and persist as IgM memory B-cells.^85^ Notably, PfSPZ vaccination indeed induces durable PfCSP-targeting IgM antibodies that efficiently fix complement and inhibit hepatocyte invasion.^64^ We hypothesize that a similar effect might exist with regard to PfMSP5, and that anti-PfMSP5-IgM could contribute to PfSPZ Vaccine-induced protection from malaria.

Given the small number of only four protected participants, associating specific antibodies or antibody profiles with sterile protection is rather elusive but might still give some insight into the role of synergising naturally acquired antibodies in a pre-exposed population. Comparing the protected subjects with the remaining unprotected study population, we indeed identified a set of antigen-specific IgG antibodies that showed correlation with protection. Notably, these did not only include the vaccine-induced PfCSP-specific antibodies but also IgG directed against PfAMA1 and PfRON2. Both antigens have been prominent vaccine candidates over the years.^86^ Regarding IgM, antibodies targeting both PfCSP and PfMSP5 showed higher levels in protected compared to unprotected individuals, although the difference was not statistically significant.

CHMI triggered a highly personalised malaria antigen specific humoral immune response amongst the study participants developing asexual blood stage parasitaemia. In placebo recipients as well as unprotected vaccinees, antibodies against a broad range of different antigens were induced. Breadth and composition of antibody profiles strongly differed between individuals but did not correlate with the HIV infection status. Highest increases in antibody reactivities were observed against known markers of malaria infection such as PfETRAMP10.2,^87^^,^^88^ reflecting the development of blood stage parasitaemia in the placebo group (0% protection rate). In contrast, both HIV positive and negative vaccinees barely reacted to any of the antigens with antibody titers rather declining over the challenge phase. In particular, no further boosting effect on the vaccination-acquired IgG and IgM antibodies directed against PfCSP and PfMSP5 was observed. The only exception is again an IgG response to PfETRAMP10.2 and IgM response to PfREX1 in HIV positive vaccinees, again marking a short-term blood stage infection in those individuals.^89^ The lack of immunogenicity of CHMI in HIV negative vaccinees is well explained by the fact that most of these volunteers were sterilely protected against CHMI (80% protection rate), indicating little exposure to blood stage infection as parasites do not egress from the liver or are immediately cleared.^90^ Previous protein microarray studies similarly found protection from both Pf and *P. vivax* infection to be associated with an attenuated response to CHMI.^29^^,^^91^ The only HIV negative vaccinee with considerably expanded antibody reactivity was indeeed unprotected (Fig. 7d). None of the HIV positive vaccinees was protected from CHMI, thus the lack of a broad humoral immune response as observed in the placebo group might be surprising. It is conceivable that immunisation might still impact on the parasite burden in unprotected vaccinees. Indeed, an extended pre-patent period and time-to-treatment was observed in unprotected vaccinees compared to placebo recipients.^26^

Our findings support similar antibody baseline profiles and comparable responses to PfSPZ vaccination and CHMI in HIV positive and negative subjects. All HIV positive participants enrolled in this trial were under antiretroviral treatment and displayed stable CD4+ lymphocyte counts >500 cells/μl, which should ameliorate the impaired immune response to novel stimuli or loss of existing immunity as observed under progressing CD4+ cell decline.^25^ Still, none of the HIV infected vaccinees were protected against CHMI, compared to 80% sterile protection in the HIV negative group. This stark difference in vaccine efficacy in the absence of clear differences in antibody patterns imply that cellular immune effector mechanisms either interacting with antibodies or on their own are essential for PfSPZ Vaccine-induced protection. Recently, a review also summarized that HIV and malaria co-infection is associated with an expansion of atypical memory B-cells as well as reduced opsonising antibodies. In addition, HIV infection is associated with dysregulated inflammatory immune response, which is potentially leading to a perturbated immune response to malaria.^92^ However, functional antibody and systems serology analysis showed post immunization sera from HIV negative vaccinees had significantly higher inhibition of PfSPZ invasion of hepatocytes *in vitro* and antibody-dependent complement deposition (ADCD) and Fcγ3B binding by anti-PfCSP and ADCD by anti–cell-traversal protein for ookinetes and SPZ (anti-PfCelTOS) antibodies.^26^

Our study has several limitations, especially the small number of samples available for analysis in a phase 1 clinical trial. Clearly separating the impacts of HIV infection, vaccination or CHMI protection status on humoral immune responses is potentially biased due to the lack of protected placebo recipients or HIV positive participants. Also, baseline antibody breadths differed between the HIV positive and negative population, even though variance in both was high. The HIV positive study population described in this manuscript was under well-controlled antiretroviral therapy, and viral concentration was below the detection limit. Not all people living with HIV are aware of the disease, take antiretroviral therapy regularly, and are able to control the viral load. In Africa, depending on the age, population-wide viral suppression was estimated between 62% and 76%, so even though the inclusion criteria of the study does not allow for the generalization of the results on the overall HIV positive population, it nevertheless is representative for a considerable proportion of the HIV-seroconverted individuals.^93^ The method of protein microarrays also comes with limitations. Measured signal intensities correlate with actual antibody levels but do not allow precise quantifications.^29^ Comparisons of signal intensities between individuals and time points thus can only be considered as rough estimates. Represented plasmodial antigens were expressed in a prokaryotic expression system, so altered protein conformations and therefore potential false-negative^29^ or even false-positive findings are likely. A rather strict definition of seropositivity thresholds in this study, intended to avoid background interference, might contribute to an underestimated effect of interventions on increase of antibody responses. The microarray used in this study comprised a limiting number of 262 fragments, representing 228 pre-selected antigens, with some of them not being full-length proteins but expressed as protein domains only. The good accordance of our findings to previous PfSPZ Vaccine studies based on a whole-proteome array^32^ yet suggests that using microarrays covering carefully down-selected Pf antigen subsets still provides meaningful insight into immunodominance and dynamics of vaccine induced responses.

In conclusion, we demonstrated that immunisation of malaria pre-exposed Tanzanian adults with PfSPZ Vaccine induces focussed IgG and IgM antibody response patterns with no obvious difference detectable between HIV positive and negative volunteers. As expected, and irrespectively of the HIV infection status, PfCSP was one of the main targets of vaccine-induced antibodies. However, PfMSP5 proved also highly immunogenic and was frequently targeted by IgM antibodies. We showed that this protein indeed seems to be expressed on the surface of PfSPZ and therefore might pose an interesting candidate for next generation pre-erythrocytic stage malaria vaccines.

## Contributors

Planning of study: CD, RF, BM.

Conduct of experiments: AY, JPD, AT, FRL, RF, SS.

Microarray design: PLF.

Access and verification of underlying data: FRL, AT, RF, CD, BM.

Analysis of data: FRL, AT, RF, CD, BM.

Supervision of study: CD, RF, PGK.

Writing of manuscript: AT, FRL, AY, CD, RF.

Clinical study conduct: MM, AT, FM, MR, GN, KR, SJ, TS, YA, BKLS, LWPC, TLR, PFB; Clinical study design: SA, SJ, LWPC, TLR, PFB, TM, SLH.

All authors read and approved the manuscript.

## Data sharing statement

Individual participant data that underlie the results reported in this publication are available from the clinical trial sponsor on the basis of a data sharing agreement on reasonable request. Deidentified data of the clinical datasets as well as the microarray is housed in ImmPort and is publicly available (accession #: SDY1909). Correspondence should be submitted to Dr. Steven L. Hoffman.

## Declaration of interests

Sanaria Inc. manufactured PfSPZ Vaccine and PfSPZ Challenge and was the sponsor of the clinical trial. YA, BKLS, TLR, TM, and SLH are salaried, full-time employees of Sanaria, and LWPC and PFB were full-time, salaried employees of Sanaria at the time the trial was conducted. All authors associated with Sanaria have potential conflicts of interest. All other authors declare that they have no competing interests.

## References

[1] World Health Organization (2013).

[2] World Health Organization (2023).

[3] Phillips M.A., Burrows J.N., Manyando C., van Huijsduijnen R.H., Van Voorhis W.C., Wells T.N.C. (2017). Malaria. Nat Rev Dis Prim.

[4] mal ERARCPoBS, Enabling T. (2017). malERA: an updated research agenda for basic science and enabling technologies in malaria elimination and eradication. PLoS Med.

[5] WHO (2022). Weekly epidemiological record, 2022, vol. 97, 09 [full issue]. Wkly Epidemiol Rec.

[6] World Health Organization (2014).

[7] Datoo M.S., Dicko A., Tinto H. (2024). Safety and efficacy of malaria vaccine candidate R21/Matrix-M in African children: a multicentre, double-blind, randomised, phase 3 trial. Lancet.

[8] Datoo M.S., Natama M.H., Somé A. (2021). Efficacy of a low-dose candidate malaria vaccine, R21 in adjuvant Matrix-M, with seasonal administration to children in Burkina Faso: a randomised controlled trial. Lancet.

[9] Osoro C.B., Ochodo E., Kwambai T.K. (2024). Policy uptake and implementation of the RTS,S/AS01 malaria vaccine in sub-Saharan African countries: status 2 years following the WHO recommendation. BMJ Glob Health.

[10] Gelband H., Carshon-Marsh R., Ansumana R. (2023). Could vaccinating adults against malaria materially reduce adult mortality in high-transmission areas?. Malar J.

[11] World Health Organization (2022).

[12] Richie T.L., Church L.W.P., Murshedkar T. (2023). Sporozoite immunization: innovative translational science to support the fight against malaria. Expert Rev Vaccines.

[13] Goswami D., Patel H., Betz W. (2024). A replication competent Plasmodium falciparum parasite completely attenuated by dual gene deletion. EMBO Mol Med.

[14] Mordmüller B., Surat G., Lagler H. (2017). Sterile protection against human malaria by chemoattenuated PfSPZ vaccine. Nature.

[15] Mwakingwe-Omari A., Healy S.A., Lane J. (2021). Two chemoattenuated PfSPZ malaria vaccines induce sterile hepatic immunity. Nature.

[16] Sulyok Z., Fendel R., Eder B. (2021). Heterologous protection against malaria by a simple chemoattenuated PfSPZ vaccine regimen in a randomized trial. Nat Commun.

[17] Luke T.C., Hoffman S.L. (2003). Rationale and plans for developing a non-replicating, metabolically active, radiation-attenuated Plasmodium falciparum sporozoite vaccine. J Exp Biol.

[18] Richie T.L., Billingsley P.F., Sim B.K. (2015). Progress with Plasmodium falciparum sporozoite (PfSPZ)-based malaria vaccines. Vaccine.

[19] Jongo S.A., Church L.W.P., Mtoro A.T. (2020). Increase of dose associated with decrease in protection against controlled human malaria infection by PfSPZ vaccine in Tanzanian adults. Clin Infect Dis.

[20] Sissoko M.S., Healy S.A., Katile A. (2017). Safety and efficacy of PfSPZ Vaccine against Plasmodium falciparum via direct venous inoculation in healthy malaria-exposed adults in Mali: a randomised, double-blind phase 1 trial. Lancet Infect Dis.

[21] Sissoko M.S., Healy S.A., Katile A. (2022). Safety and efficacy of a three-dose regimen of Plasmodium falciparum sporozoite vaccine in adults during an intense malaria transmission season in Mali: a randomised, controlled phase 1 trial. Lancet Infect Dis.

[22] Subramaniam K.S., Skinner J., Ivan E. (2015). HIV malaria Co-infection is associated with atypical memory B cell expansion and a reduced antibody response to a broad array of Plasmodium falciparum antigens in Rwandan adults. PLoS One.

[23] Dwyer-Lindgren L., Cork M.A., Sligar A. (2019). Mapping HIV prevalence in sub-Saharan Africa between 2000 and 2017. Nature.

[24] Otieno L., Guerra Mendoza Y., Adjei S. (2020). Safety and immunogenicity of the RTS,S/AS01 malaria vaccine in infants and children identified as HIV-infected during a randomized trial in sub-Saharan Africa. Vaccine.

[25] Crum-Cianflone N.F., Wallace M.R. (2014). Vaccination in HIV-infected adults. AIDS Patient Care STDS.

[26] Jongo S., Church L.W.P., Milando F. (2024). Safety and protective efficacy of PfSPZ Vaccine administered to HIV-negative and -positive Tanzanian adults. J Clin Invest.

[27] Liang L., Felgner P.L. (2015). A systems biology approach for diagnostic and vaccine antigen discovery in tropical infectious diseases. Curr Opin Infect Dis.

[28] Crompton P.D., Kayala M.A., Traore B. (2010). A prospective analysis of the Ab response to Plasmodium falciparum before and after a malaria season by protein microarray. Proc Natl Acad Sci U S A.

[29] Doolan D.L., Mu Y., Unal B. (2008). Profiling humoral immune responses to P. falciparum infection with protein microarrays. Proteomics.

[30] Felgner P.L., Roestenberg M., Liang L. (2013). Pre-erythrocytic antibody profiles induced by controlled human malaria infections in healthy volunteers under chloroquine prophylaxis. Sci Rep.

[31] Helb D.A., Tetteh K.K., Felgner P.L. (2015). Novel serologic biomarkers provide accurate estimates of recent Plasmodium falciparum exposure for individuals and communities. Proc Natl Acad Sci U S A.

[32] Camponovo F., Campo J.J., Le T.Q. (2020). Proteome-wide analysis of a malaria vaccine study reveals personalized humoral immune profiles in Tanzanian adults. Elife.

[33] Wichers J.S., Tonkin-Hill G., Thye T. (2021). Common virulence gene expression in adult first-time infected malaria patients and severe cases. Elife.

[34] Obiero J.M., Campo J.J., Scholzen A. (2019). Antibody biomarkers associated with sterile protection induced by controlled human malaria infection under chloroquine prophylaxis. mSphere.

[35] Dent A.E., Nakajima R., Liang L. (2015). Plasmodium falciparum protein microarray antibody profiles correlate with protection from symptomatic malaria in Kenya. J Infect Dis.

[36] Ibanez J., Fendel R., Lorenz F.R. (2022). Efficacy, T cell activation and antibody responses in accelerated Plasmodium falciparum sporozoite chemoprophylaxis vaccine regimens. NPJ Vaccines.

[37] King C.L., Davies D.H., Felgner P. (2015). Biosignatures of exposure/transmission and immunity. Am J Trop Med Hyg.

[38] Kobayashi T., Jain A., Liang L. (2019). Distinct antibody signatures associated with different malaria transmission intensities in Zambia and Zimbabwe. mSphere.

[39] de Jong S.E., van Unen V., Manurung M.D. (2021). Systems analysis and controlled malaria infection in Europeans and Africans elucidate naturally acquired immunity. Nat Immunol.

[40] Taghavian O., Jain A., Joyner C.J. (2018). Antibody profiling by proteome microarray with multiplex isotype detection reveals overlap between human and Aotus nancymaae controlled malaria infections. Proteomics.

[41] R Core Team (2019).

[42] Ritchie M.E., Silver J., Oshlack A. (2007). A comparison of background correction methods for two-colour microarrays. Bioinformatics.

[43] Ritchie M.E., Phipson B., Wu D. (2015). Limma powers differential expression analyses for RNA-sequencing and microarray studies. Nucleic Acids Res.

[44] Silver J.D., Ritchie M.E., Smyth G.K. (2009). Microarray background correction: maximum likelihood estimation for the normal-exponential convolution. Biostatistics.

[45] Wobbrock J.O., Findlater L., Gergle D., Higgins J.J. (2011). Proceedings of the SIGCHI conference on human factors in computing systems.

[46] Wickham H. (2016).

[47] Warnes G.R., Bolker B., Bonebakker L. (2009). gplots: various R programming tools for plotting data. R Package Version.

[48] Clarke E., Sherrill-Mix S. (2017). https://CRANR-project.org.

[49] Edwards S. (2019).

[50] Turewicz M., Ahrens M., May C., Marcus K., Eisenacher M. (2016). PAA: an R/bioconductor package for biomarker discovery with protein microarrays. Bioinformatics.

[51] Borrmann S., Sulyok Z., Müller K. (2020). Mapping of safe and early chemo-attenuated live *Plasmodium falciparum* immunization identifies immune signature of vaccine efficacy. bioRxiv.

[52] Tibúrcio M., Yang A.S.P., Yahata K. (2019). A novel tool for the generation of conditional knockouts to study gene function across the Plasmodium falciparum life cycle. mBio.

[53] Miyazaki S., Yang A.S.P., Geurten F.J.A. (2020). Generation of novel Plasmodium falciparum NF135 and NF54 lines expressing fluorescent reporter proteins under the control of strong and constitutive promoters. Front Cell Infect Microbiol.

[54] Baum E., Sattabongkot J., Sirichaisinthop J. (2016). Common asymptomatic and submicroscopic malaria infections in Western Thailand revealed in longitudinal molecular and serological studies: a challenge to malaria elimination. Malar J.

[55] Yang A.S.P., van Waardenburg Y.M., van de Vegte-Bolmer M. (2021). Zonal human hepatocytes are differentially permissive to Plasmodium falciparum malaria parasites. EMBO J.

[56] Kwenti T.E. (2018). Malaria and HIV coinfection in sub-Saharan Africa: prevalence, impact, and treatment strategies. Res Rep Trop Med.

[57] Naing C., Sandhu N.K., Wai V.N. (2016). The effect of malaria and HIV Co-infection on anemia: a meta-analysis. Medicine (Baltim).

[58] Patnaik P., Jere C.S., Miller W.C. (2005). Effects of HIV-1 serostatus, HIV-1 RNA concentration, and CD4 cell count on the incidence of malaria infection in a cohort of adults in rural Malawi. J Infect Dis.

[59] Whitworth J., Morgan D., Quigley M. (2000). Effect of HIV-1 and increasing immunosuppression on malaria parasitaemia and clinical episodes in adults in rural Uganda: a cohort study. Lancet.

[60] Hoffman S.L., Goh L.M., Luke T.C. (2002). Protection of humans against malaria by immunization with radiation-attenuated Plasmodium falciparum sporozoites. J Infect Dis.

[61] Seder R.A., Chang L.J., Enama M.E. (2013). Protection against malaria by intravenous immunization with a nonreplicating sporozoite vaccine. Science.

[62] Kumar K.A., Sano G., Boscardin S. (2006). The circumsporozoite protein is an immunodominant protective antigen in irradiated sporozoites. Nature.

[63] Epstein J.E., Tewari K., Lyke K.E. (2011). Live attenuated malaria vaccine designed to protect through hepatic CD8⁺ T cell immunity. Science.

[64] Zenklusen I., Jongo S., Abdulla S. (2018). Immunization of malaria-Preexposed volunteers with PfSPZ vaccine elicits long-lived IgM invasion-inhibitory and complement-fixing antibodies. J Infect Dis.

[65] Marshall V.M., Tieqiao W., Coppel R.L. (1998). Close linkage of three merozoite surface protein genes on chromosome 2 of Plasmodium falciparum. Mol Biochem Parasitol.

[66] Wu T., Black C.G., Wang L., Hibbs A.R., Coppel R.L. (1999). Lack of sequence diversity in the gene encoding merozoite surface protein 5 of Plasmodium falciparum. Mol Biochem Parasitol.

[67] Rainczuk A., Smooker P.M., Kedzierski L., Black C.G., Coppel R.L., Spithill T.W. (2003). The protective efficacy of MSP4/5 against lethal Plasmodium chabaudi adami challenge is dependent on the type of DNA vaccine vector and vaccination protocol. Vaccine.

[68] Kedzierski L., Black C.G., Goschnick M.W., Stowers A.W., Coppel R.L. (2002). Immunization with a combination of merozoite surface proteins 4/5 and 1 enhances protection against lethal challenge with Plasmodium yoelii. Infect Immun.

[69] Perraut R., Joos C., Sokhna C. (2014). Association of antibody responses to the conserved Plasmodium falciparum merozoite surface protein 5 with protection against clinical malaria. PLoS One.

[70] Medeiros M.M., Fotoran W.L., dalla Martha R.C., Katsuragawa T.H., Pereira da Silva L.H., Wunderlich G. (2013). Natural antibody response to Plasmodium falciparum merozoite antigens MSP5, MSP9 and EBA175 is associated to clinical protection in the Brazilian Amazon. BMC Infect Dis.

[71] Wilson K.L., Pouniotis D., Hanley J. (2019). A synthetic nanoparticle based vaccine approach targeting MSP4/5 is immunogenic and induces moderate protection against murine blood-stage malaria. Front Immunol.

[72] Sanders P.R., Kats L.M., Drew D.R. (2006). A set of glycosylphosphatidyl inositol-anchored membrane proteins of Plasmodium falciparum is refractory to genetic deletion. Infect Immun.

[73] Epstein J.E., Paolino K.M., Richie T.L. (2017). Protection against Plasmodium falciparum malaria by PfSPZ vaccine. JCI Insight.

[74] Macià D., Campo J.J., Moncunill G. (2022). Strong off-target antibody reactivity to malarial antigens induced by RTS,S/AS01E vaccination is associated with protection. JCI Insight.

[75] Dobaño C., Ubillos I., Jairoce C. (2019). RTS,S/AS01E immunization increases antibody responses to vaccine-unrelated Plasmodium falciparum antigens associated with protection against clinical malaria in African children: a case-control study. BMC Med.

[76] Le Roch K.G., Zhou Y., Blair P.L. (2003). Discovery of gene function by expression profiling of the malaria parasite life cycle. Science.

[77] Lindner S.E., Swearingen K.E., Shears M.J. (2019). Transcriptomics and proteomics reveal two waves of translational repression during the maturation of malaria parasite sporozoites. Nat Commun.

[78] Zanghì G., Vembar S.S., Baumgarten S. (2018). A specific PfEMP1 is expressed in P. Falciparum sporozoites and plays a role in hepatocyte infection. Cell Rep.

[79] Yang A.S.P., O'Neill M.T., Jennison C. (2017). Cell traversal activity is important for Plasmodium falciparum liver infection in humanized mice. Cell Rep.

[80] Lin C.S., Uboldi A.D., Epp C. (2016). Multiple Plasmodium falciparum merozoite surface protein 1 complexes mediate merozoite binding to human erythrocytes. J Biol Chem.

[81] Boyle M.J., Chan J.A., Handayuni I. (2019). IgM in human immunity to Plasmodium falciparum malaria. Sci Adv.

[82] Kurtovic L., Behet M.C., Feng G. (2018). Human antibodies activate complement against Plasmodium falciparum sporozoites, and are associated with protection against malaria in children. BMC Med.

[83] Mordmüller B., Sulyok Z., Sulyok M. (2022). A PfSPZ vaccine immunization regimen equally protective against homologous and heterologous controlled human malaria infection. NPJ Vaccines.

[84] Tan J., Sack B.K., Oyen D. (2018). A public antibody lineage that potently inhibits malaria infection through dual binding to the circumsporozoite protein. Nat Med.

[85] Krishnamurty A.T., Thouvenel C.D., Portugal S. (2016). Somatically hypermutated plasmodium-specific IgM(+) memory B cells are rapid, plastic, early responders upon malaria rechallenge. Immunity.

[86] Srinivasan P., Ekanem E., Diouf A. (2014). Immunization with a functional protein complex required for erythrocyte invasion protects against lethal malaria. Proc Natl Acad Sci U S A.

[87] van den Hoogen L.L., Walk J., Oulton T. (2019). Antibody responses to antigenic targets of recent exposure are associated with low-density parasitemia in controlled human *Plasmodium falciparum* infections. Front Microbiol.

[88] Helba D.A., Tetteh K.K.A., Felgner P.L. (2015). Novel serologic biomarkers provide accurate estimates of recent *Plasmodium falciparum* exposure for individuals and communities. Proc Natl Acad Sci U S A.

[89] Tadesse F.G., Lanke K., Nebie I. (2017). Molecular markers for sensitive detection of Plasmodium falciparum asexual stage parasites and their application in a malaria clinical trial. Am J Trop Med Hyg.

[90] Lell B., Mordmüller B., Dejon Agobe J.C. (2018). Impact of sickle cell trait and naturally acquired immunity on uncomplicated malaria after controlled human malaria infection in adults in Gabon. Am J Trop Med Hyg.

[91] Arévalo-Herrera M., Lopez-Perez M., Dotsey E. (2016). Antibody profiling in naïve and semi-immune individuals experimentally challenged withPlasmodium *vivax* sporozoites. PLoS Negl Trop Dis.

[92] Figueroa-Romero A., Saura-Lázaro A., Fernández-Luis S., González R. (2024). Uncovering HIV and malaria interactions: the latest evidence and knowledge gaps. Lancet HIV.

[93] Green D., Tordoff D.M., Kharono B. (2020). Evidence of sociodemographic heterogeneity across the HIV treatment cascade and progress towards 90-90-90 in sub-Saharan Africa–a systematic review and meta-analysis. J Int AIDS Soc.

