# Supplementary material for: PfSPZ Vaccine induces focused humoral immune response in HIV positive and negative Tanzanian adults
Source: eBioMedicine. 2024 Sep 30;108:105364. doi: 10.1016/j.ebiom.2024.105364 (PMC11464252; doi:10.1016/j.ebiom.2024.105364)
Supplement: Supplementary Figures [file mmc1.docx]

**Supplementary material**


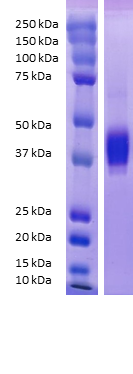


# Supplementary Figure 1: Expression of recombinant MSP5.

Recombinant PfMSP5 protein was expressed in HEK 293 F cells and subsequently purified using Ni-agarose affinity chromatography. The purity of the protein was assessed using a gradient SDS-PAGE and stained using Coomassie. Marker: Color Prestained Protein Standard, Broad Range (NEB).


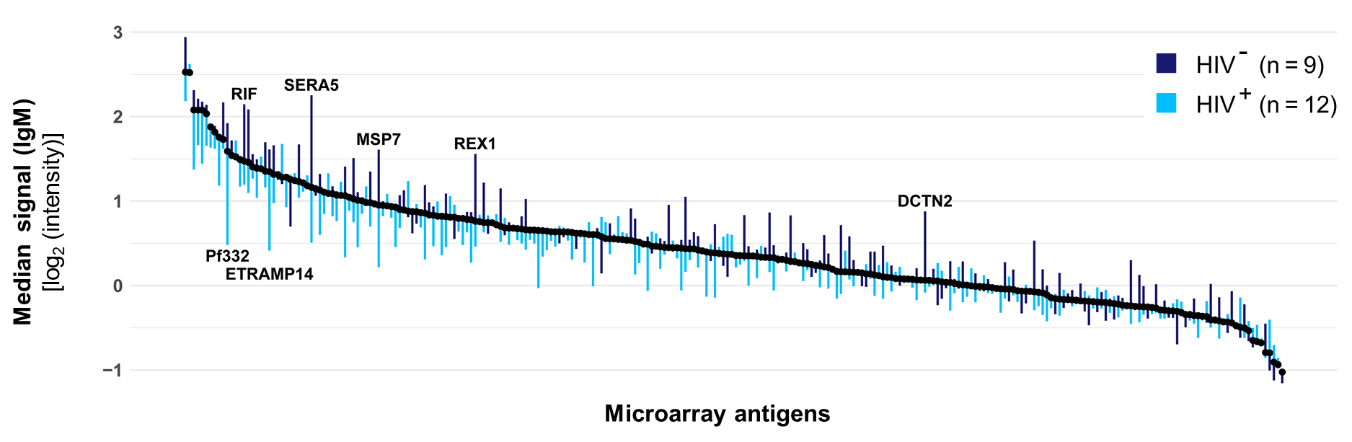


# Supplementary Figure 2: Baseline IgM immunity of study population stratified by HIV infection status.

Antigens are sorted according to their overall median IgM signal intensity, weighted for the different number of subjects in the HIV positive and HIV negative group. Bars give the deviation of the median signal intensities in the HIV negative (dark blue) and HIV positive group (light blue) from the overall median.


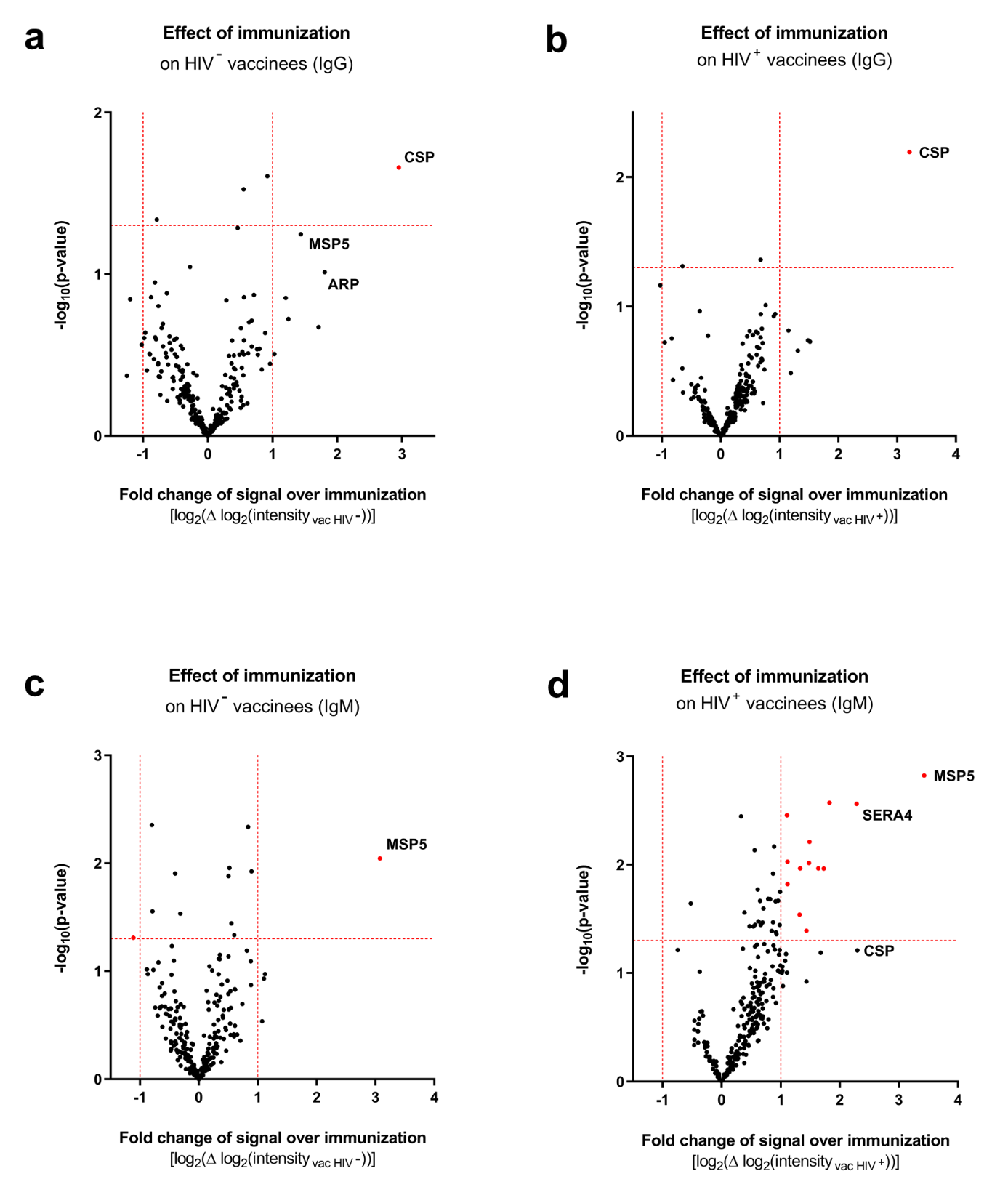


# Supplementary Figure 3: Effect of immunization stratified by HIV status.

Volcano plots of fold change in IgG (a, b) or IgM (c, d) and p-values (paired Student´s t-test) of average signal intensity in HIV negative (a, c; n = 5) or HIV positive vaccinees (b, d; n = 6) compared to their baseline for all microarray antigens. Differentially recognized antigens (p-value < 0.05 and fold change > 2) are depicted in red (see Supplementary Table 2 for antigen abbreviations).


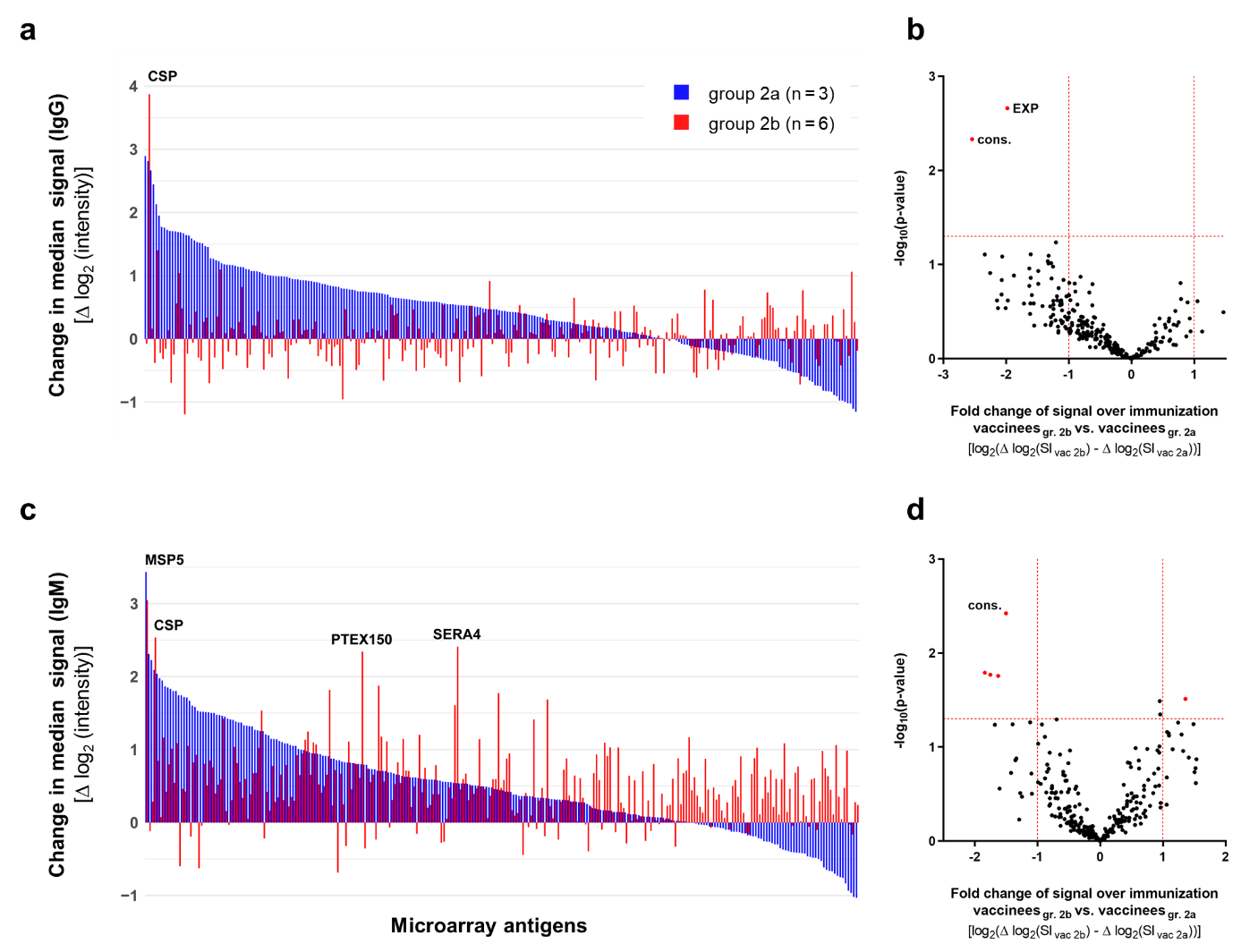


# Supplementary Figure 4: Effect of vaccine doses in HIV positive vaccinees.

To identify vaccination-induced antibodies, microarray reactivity in samples collected after the completed immunization phase were compared to their individual baseline reactivity. HIV positive vaccinees received a cumulative dose of either 2.25 × 10^6^ (group 2a) or 4.5 × 10^6^ PfSPZ of PfSPZ Vaccine (group 2b). (a, c) Median changes in IgG (a) and IgM (c) signal intensities across 262 microarray antigens over the immunization phase were assessed amongst group 2a (n = 3) and group 2b vaccinees (n = 6). Antigens are sorted by median intensity changes in the group 2a. (b, d) Volcano plots of mean changes in microarray IgG (b) and IgM (d) signal intensities over immunization in the group 2b vaccinees compared to the group 2a vaccinees. Fold change and p-values (Welch-corrected Student´s t-test) are given for all microarray antigens. Significantly differentially recognized reactive antigens (p-value < 0.05 and fold change > 2) are depicted in red (see Supplementary Table 2 for antigen abbreviations).


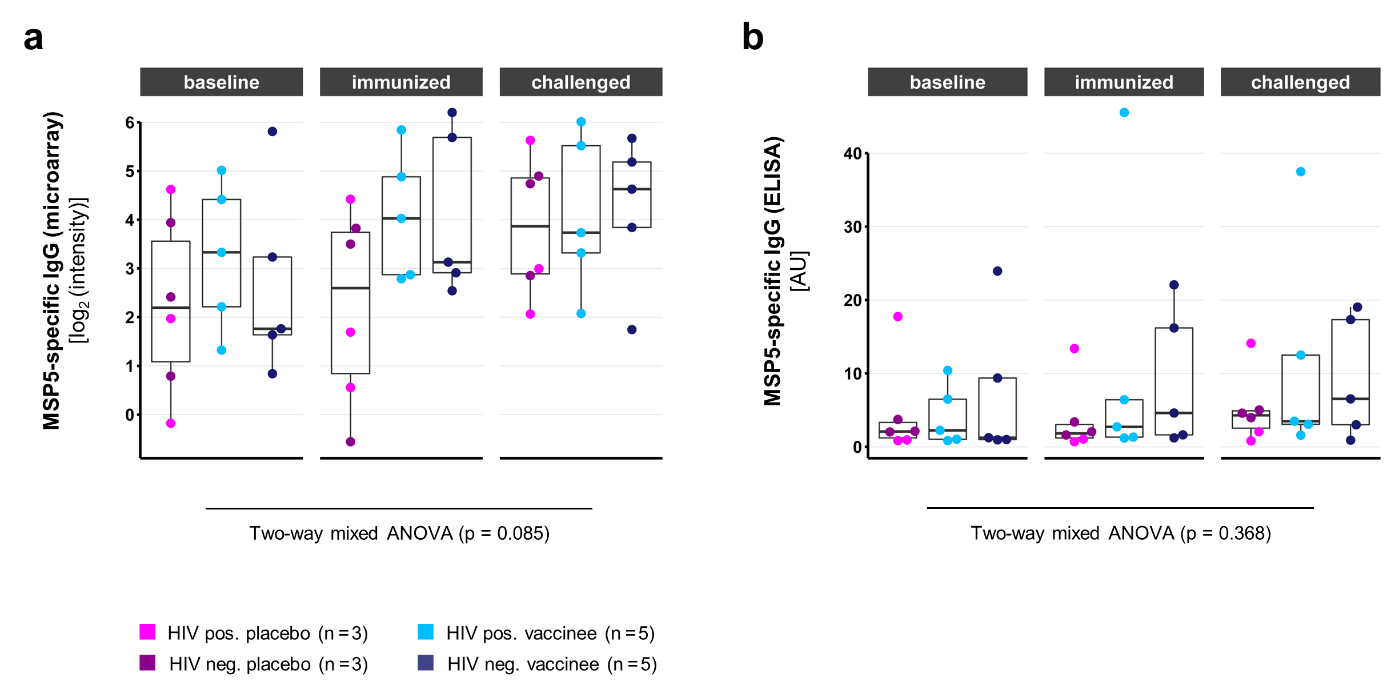


# Supplementary Figure 5: Antibody kinetic of PfMSP5-specific IgG.

Individual microarray signal intensities (a) and antibody titres measured by ELISA (b) are compared at baseline, 14 days after immunization and 28 days after challenge. The influence of intervention group (placebo, vaccine) and sampling time point (baseline, immunized, challenged) on the measured signal intensity or titre was evaluated using a two-way mixed ANOVA model. The boxplots give median antibody breadths, interquartile ranges (IQR) and whiskers of length 1.5 × IQR.


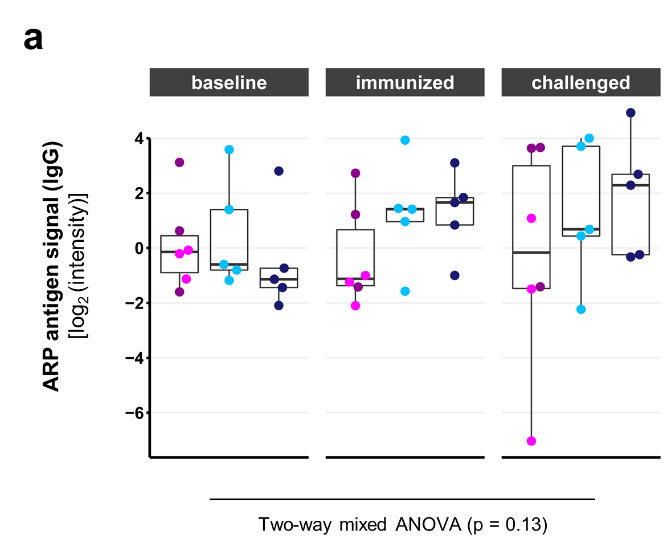


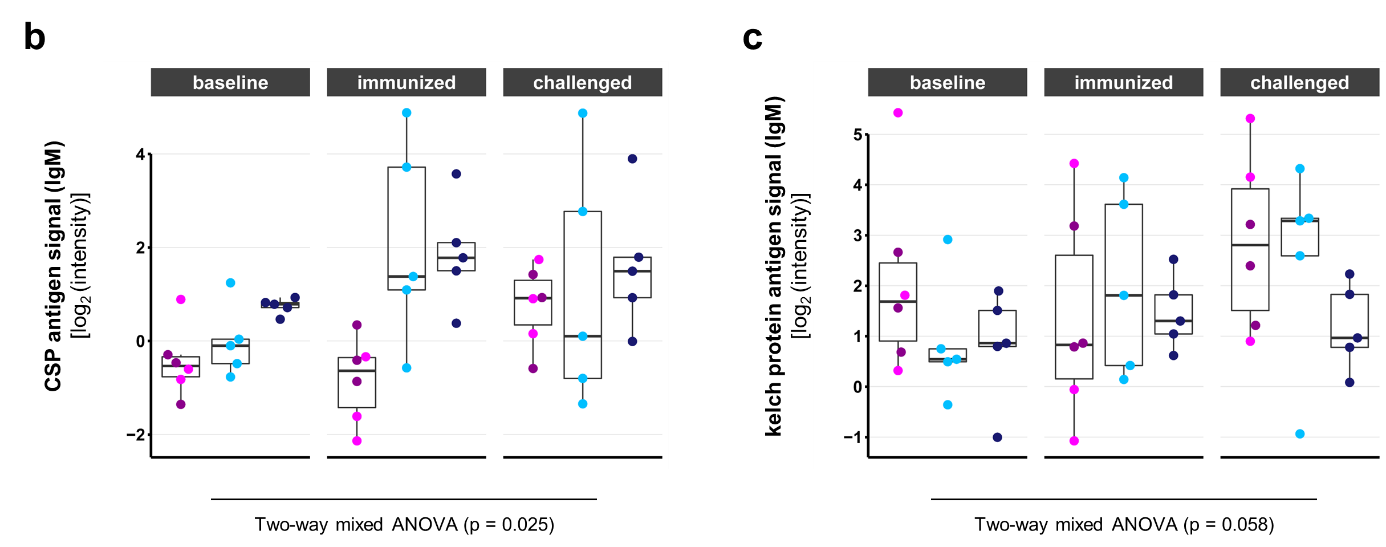


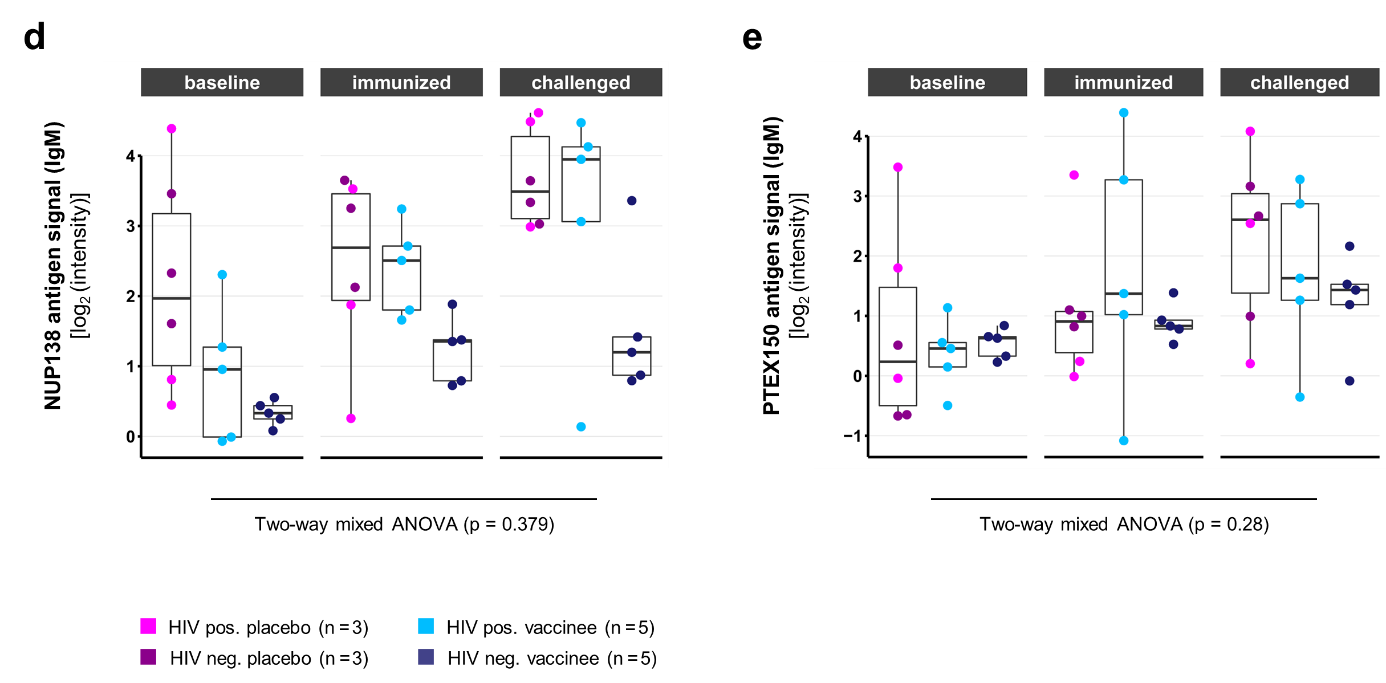


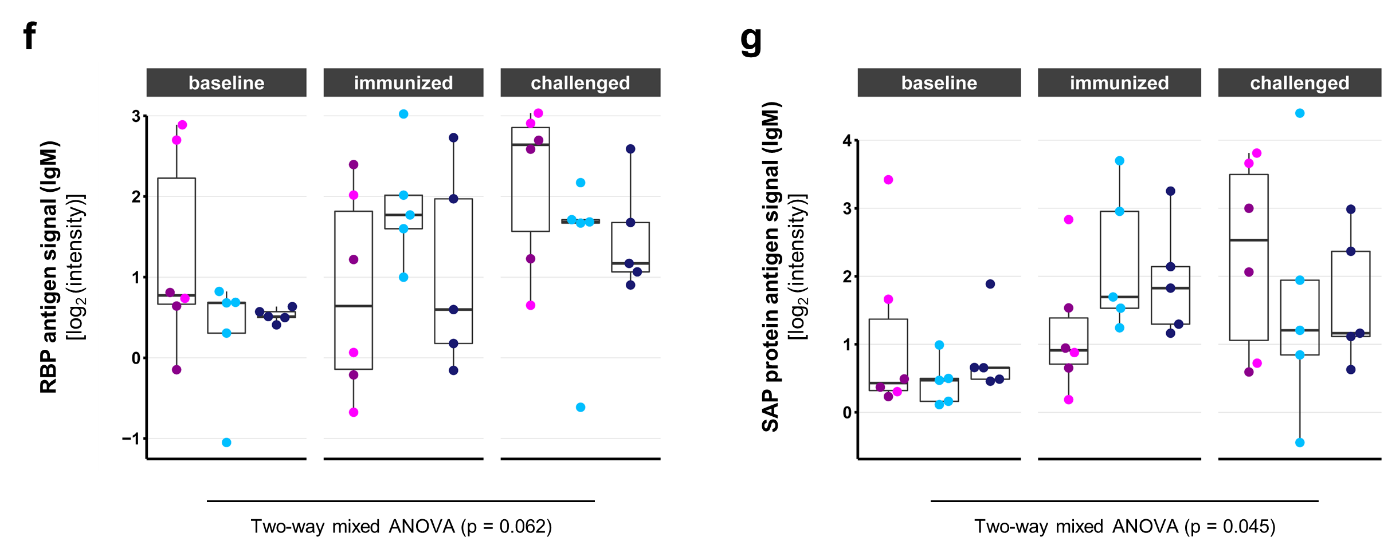


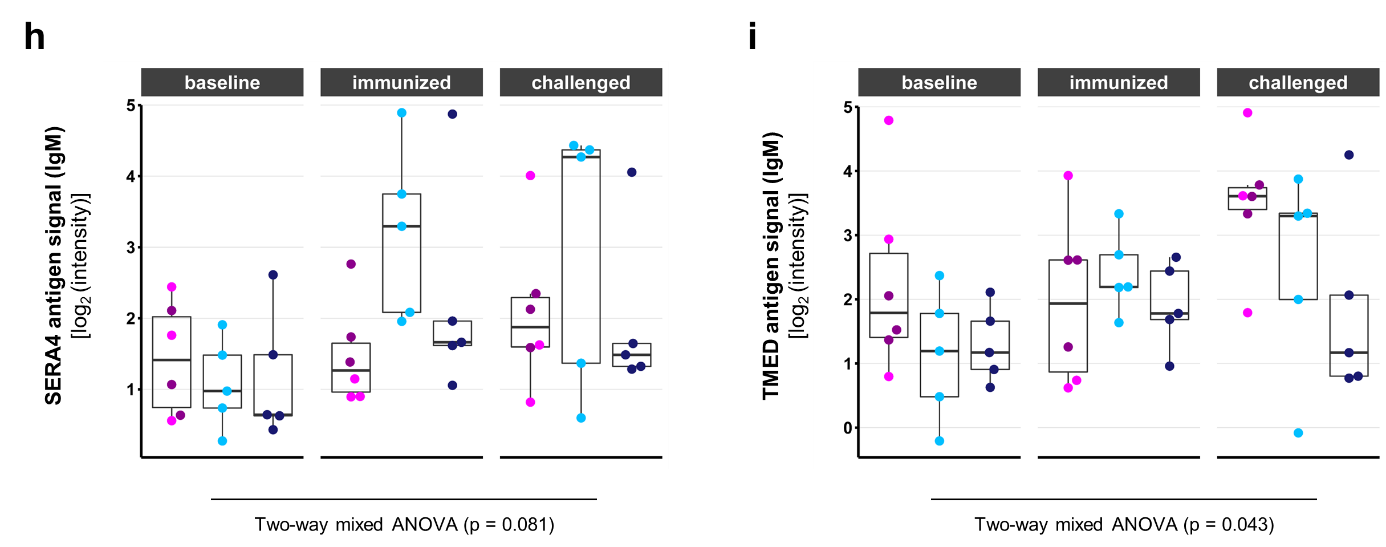


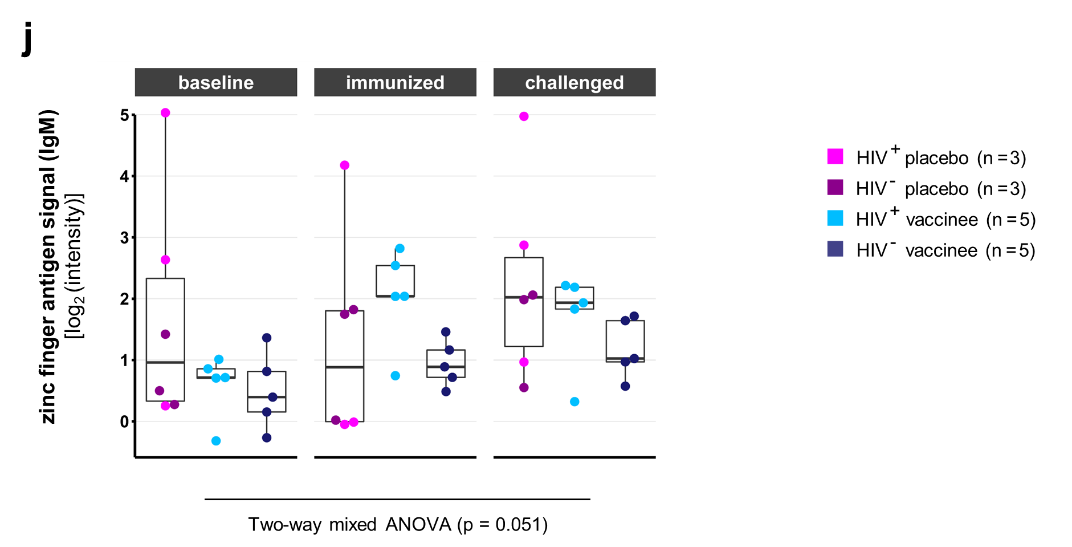


# Supplementary Figure 6: Antibody kinetics of vaccine-induced antibodies.

Individual IgG (a) and IgM (b-j) microarray signal intensities of vaccination-induced antibodies (see Fig. 3) are compared at baseline, 14 days after immunization and 28 days after challenge. The influence of intervention group (placebo, vaccine) and sampling time point (baseline, immunized, challenged) on the measured microarray antigen signal intensity was evaluated using a two-way mixed ANOVA model. The boxplots give median antibody breadths, interquartile ranges (IQR) and whiskers of length 1.5 × IQR.
